# Perspectives on exercise intensity, volume, step characteristics and health outcomes in walking for transport

**DOI:** 10.3389/fpubh.2022.911863

**Published:** 2022-10-21

**Authors:** Peter Schantz, Karin Sofia Elisabeth Olsson, Jane Salier Eriksson, Hans Rosdahl

**Affiliations:** ^1^The Research Unit for Movement, Health and Environment, Department of Physical Activity and Health, The Swedish School of Sport and Health Sciences, GIH, Stockholm, Sweden; ^2^Section of Sustainable Health, Department of Public Health and Clinical Medicine, Umeå University, Umeå, Sweden; ^3^The Research Unit for Movement, Health and Environment, Department of Physiology, Nutrition and Biomechanics, The Swedish School of Sport and Health Sciences, GIH, Stockholm, Sweden

**Keywords:** commuter walking, exercise intensity, energy expenditure, metabolic equivalent of task, trip duration, trip frequency, rated perceived exertion, cycling

## Abstract

**Background:**

Quantification of movement intensity and energy utilization, together with frequency of trips, duration, distance, step counts and cadence, is essential for interpreting the character of habitual walking for transport, and its potential support of health. The purpose of the study is to illuminate this with valid methods and novel perspectives, and to thereby provide a new basis for characterizing and interpreting walking in relation to health outcomes.

**Methods:**

Habitual middle-aged commuting pedestrians (males = 10, females = 10) were investigated in the laboratory at rest and with maximal treadmill and cycle ergometer tests. Thereafter, levels of oxygen uptake, energy expenditure, ventilation, heart rate, blood lactate, rated perceived exertion, cadence, number of steps, duration, distance, and speed were recorded during the normal walking commute of each participant in Greater Stockholm, Sweden. The number of commutes per week over the year was self-reported.

**Results:**

Walking in the field demanded about 30% more energy per km compared to level treadmill walking. For both sexes, the walking intensity in field was about 46% of maximal oxygen uptake, and energy expenditure amounted to 0.96 kcal · kg^−^^1^ · km^−^^1^. The MET values (males: 6.2; females: 6.5) mirrored similar levels of walking speed (males: 5.7; females: 5.9 km · h^−^^1^) and levels of oxygen uptake (males: 18.6; females: 19.5 mL · kg^−^^1^ · min^−^^1^). The average number of MET-hours per week in a typical month was 22 for males and 20 for females. This resulted in a total weekly energy expenditure of ~1,570 and 1,040 kcal for males and females, respectively. Over the year, the number of walking commutes and their accumulated distance was ~385 trips and 800 km for both sexes.

**Conclusion:**

Walking in naturalistic field settings demands its own studies. When males and females walk to work, their relative aerobic intensities and absolute energy demands for a given distance are similar. It is equivalent to the lower part of the moderate relative intensity domain. The combination of oxygen uptake, trip duration and frequency leads to high and sustained levels of MET-hours as well as energy expenditure per week over the year, with a clear health enhancing potential. Based on this study we recommend 6000 transport steps per day, or equivalent, during five weekdays, over the year, in order to reach optimal health gains.

## Introduction

For millions of years, walking has been a major source of physical activity for mankind. In our times, thousands of billions of steps are taken globally every day all year around. Given their normality in everyday life, these steps have long been viewed as too trivial to study.

For more than two decades this attitude has changed step by step. The recognition, in 1996, of moderate exercise intensities for gaining health effects by the U.S. Department of Health and Human Services (1) as well as the review “Walking to Health” (2) have been considered important for this change of attitude (3). The invention of step counters and accelerometry possibly enhanced the mind shift, and clearly enabled the broad scientific development that followed.

Today a number of studies exist of e.g. daily step counts, and how they relate to various health outcomes [e.g., (4–6)]. These studies are valuable, especially as nowadays steps are easy to count, and the related public health messages are simple to understand.

However, from an analytical perspective, the total number of steps per day represent such a diverse entity that it does not further our understanding of the character of the physical activity that evokes health effects. During a day, steps can vary greatly in terms of number and duration of episodes with steps, number of steps, cadence, step length, speeds (7), and the topography as well as the surface on which the walking takes place. Furthermore, even at given values these steps may represent clear differences in relative exercise intensities depending on e.g. sex, age and body weight [(8), p. 330–341]. This raises concerns as to whether, for example, walking at a certain cadence represents a moderate exercise intensity associated with health effects (9–11).

The steps taken during a day serve a number of purposes, and can be undertaken in our homes, at work, for shopping, recreation or physical exercise and for transporting ourselves from an origin to a destination. To further the understanding of one common context of walking during a day, this study aims to describe walking for transport in new ways by studying groups of pedestrians who habitually walk between their homes and work or study place.

Walking commuting is a very common form of physical activity. However, little is known about its characteristics in terms of, for example, movement intensities, durations and frequencies of trips *per se*, as well as a combination of these three. With such data, the understanding of the role commuter walking can have on certain outcomes, such as health, aerobic fitness, and weight control will increase.

Commuting to and from the workplace most often represents a regular need of transportation over the year. It can thereby be linked with considerable levels of physical activity in a year (12), and is therefore of clear interest to study further. This is even more relevant since two recent epidemiological studies have indicated that walking, as compared to cycle commuting, may lead to less health effects in certain diseases (13, 14).

We have previously described the exercise physiology of cycle commuting in Greater Stockholm, Sweden, with valid methods (15–18), and combined it with data on trip durations and frequencies (19). Commuter cycling was thereby illuminated through a broad array of relevant health related variables, such as aerobic exercise intensities, ventilation, heart rate (HR), metabolic equivalent of task (MET) levels, energy cost, perceived exertion, distances, durations, speeds, and trips per week over the year.

To our knowledge, no corresponding study exists of habitual walking commuting. Given the background stated above, and to enable comparisons with cycling, it was considered important to use the same valid methodology to study normal walking commutes of both males and females who habitually walk to work in the same overall area as we studied commuter cycling in Schantz et al. (19). Participants were studied in the laboratory and during their individual walking commutes. This allows us to describe the exercise physiology of commuter walking in new ways and enable comparisons between males and females. For that purpose, we have also compared step characteristics and energy demands during walking in the field with level treadmill walking in a range of velocities. Additionally, by combining the frequency and duration of trips over the year with their physiological measures, we have devised a set of data that allows comparisons of outcomes in other studies concerning physical activity and aerobic capacity as well as other health parameters. It does also permit us to calculate and suggest weekly levels of transport steps in order to gain optimal health effects. Finally, a basis for comparing walking and cycle commuting was also created.

## Materials and methods

The study is part of the “Physically Active Commuting in Greater Stockholm (PACS)”-project at the Åstrand Laboratory and the Laboratory of Applied Sport Science of GIH in Stockholm, Sweden. Given that one of the purposes with this study is to be able to compare the findings with those of commuter cycling, we have, as much as possible, been using the same methodology in this study as compared to Schantz et al. (19), who studied commuter cycling. We have therefore reused several text sections regarding methods from Schantz et al. (19).

### Participants

#### Recruitment of participants

10 males and 10 females were, in several steps, selected from a greater sample of active commuters. The first step in this process was requesting participants *via* advertisements in two main Stockholm morning newspapers in May and June 2004. The inclusion criteria required being a minimum of 20 years old, living in the County of Stockholm, (except for in the Municipality of Norrtälje), at least once a year walking or cycling the whole way, and of any distance to one's work or place of study. This first step of recruitment resulted in 2148 volunteers.

A paper questionnaire (The Physically Active Commuting in Greater Stockholm Questionnaire 1; PACS Q1) was sent home to the volunteers. Only the questions relevant for selecting our participants were used. This included gender, age, how physically strenuous their professional jobs were, as well as commuting frequencies per week for each month of the year and commuting time. The commuting distance based on routes drawn in maps by each respondent was also used (15). For details on the recruitment process as well as the questionnaire used, see Stigell and Schantz (12), Stigell [(20), p. 67] and the supplementary material in Schantz et al. (21).

Our sample was selected from the single mode pedestrian category (*n* = 277), i.e., those subjects who only walked, and never cycled, to work. Other criteria were that they had ages and route distances close to the median values of the male and female pedestrians, respectively (12). They also rated their daily professional jobs as light or very light physically. Letters describing the physiological studies, the test procedures, and a health declaration [for details, see (19)] were sent to the pedestrians who fulfilled the criteria. The right to terminate the tests at any time, and without having to stipulate a reason, was emphasized in the letter. A signed informed consent of participation and the health declaration was returned.

Based on this information, individuals with invalid route distances as well as with high blood pressure, or on medication that could affect normal HR were excluded. The remaining individuals were contacted by telephone to answer any potential questions, and to book test times. Telephone contacts continued until we had 10 females and 10 males who fulfilled the criteria and were willing to participate (Table 1).

**Table 1:** Characteristics of the walking commuters (mean and standard deviation, SD).

**Walking commuters**	**Age**	**Height**	**Weight**	**Body mass index**	**Heart rate** **rest**	**Systolic blood pressure**	**Diastolic blood pressure**
	**years**	**m**	**kg**	**kg** ·**m**^−^^2^	**beats** ·**min**^−^^1^	**mm Hg**	**mm Hg**
Men (*n* = 10)	**46.4** 9.5	**1.79** 0.07	**80.5** 11.9	**25.0** 3.0	**74.0** 7.0	**118** 10	**79.1** 8.3
Women (*n* = 10)	**44.0** 4.7	1.68^*^ 0.04	61.1^*^ 8.3	21.5^*^ 2.4	60.8^*^ 8.7	**109** 12	70.4^*^ 9.4

* Significant differences (p < 0.05) between men and women.

### Equipment and preparation

#### Stationary metabolic gas analysis system

For all metabolic measurements in the laboratory, we used a stationary metabolic system (SMS) (Oxycon Pro^®^, JLAB version 4.53, Carefusion GmbH, Hoechberg, Germany). Expired air was sampled continuously from the mixing chamber through a nafion tubing that connects to a nafion tubing on the inside of the equipment, and that terminates at the oxygen and carbon dioxide analyzer inlets. A digital volume transducer (DVT) at the outlet of the mixing chamber measured the ventilation (VE). HR was recorded and averaged every 15 s *via* a Polar transmitter (see below). In accordance with the manufacturer's recommendations, we switched on the equipment 30 minutes before data collection and calibrated it immediately before and after each test using built-in automated procedures. A high precision gas of 15.00% O_2_, and 6.00% CO_2_ (accuracy: O_2_ ± 0.04% rel. and CO_2_ ± 0.1% rel. Air Liquid AB, Kungsängen, Sweden) was used for calibration of the gas analyzers. A facemask with non-rebreathing air inlet valves (Combitox, Dräger Safety, Lübeck, Germany) was used and carefully fitted on the subject.

#### Mobile metabolic gas analysis system

For the collection of the metabolic variables in the field we used a mobile metabolic system (MMS) (Oxycon Mobile, JLAB version 5.10., CareFusion GmbH, Hoechberg, Germany). It had been carefully validated, as described in detail elsewhere (16–18). The MMS was assembled and switched on at a minimum of 30 min before each test. Immediately before and after each field measurement, a calibration was undertaken using built-in automated procedures. The calibrations were performed in the same environmental settings as the field measurements and enabled a check of any potential drift during the data collection. Before each measurement was started, a check phase of about 2 min was used to confirm that a recording was being made. Gas exchange and ventilation variables were measured breath by breath and averaged over 15 s for further analyses. Two differently sized facemasks (Combitox, Dräger Safety, Lübeck, Germany) were used with the MMS, and were carefully fitted on the subject.

#### Ergometer cycle and treadmill

A manually braked pendulum ergometer cycle (828E Monark Exercise AB, Vansbro, Sweden) was used. Before each measurement, the scale was zeroed with each subject sitting upright on the saddle with the feet resting on the frame between the pedals, and with the hands resting on the handle bars. The saddle height was adjusted so that the participant's knees were slightly flexed when the feet were on the pedals in their lowest position. The handle bars were adjusted to allow the participants to sit in an upright position. A digital metronome (DM70 Seiko S-Yard Co. Ltd, Tokyo, Japan) helped the subjects to maintain the correct cadence while cycling. The workload was controlled every minute by checking the cadence of the participant, and the braking force, as indicated on the pendulum scale. A treadmill (Model RL2500E, Rodby Innovation AB, Vänge, Hagby, Sweden) was also used for measurements while running.

#### Heart rate and blood pressure

HR was measured during exercise, averaged per 15 s and stored, using a Polar S610i heart rate monitor with a Polar Wearlink 31 transmitter (Polar Electro Oy, Kempele, Finland). HR was also stored and averaged per every 15 s with the MMS and the SMS. Blood pressure was measured with a manual sphygmomanometer.

#### Blood lactate sampling and analyses

Blood samples (20 μL) for lactate analysis were taken from a fingertip, and immediately transferred to a tube containing haemolysing solution. All samples were analyzed using a BIOSEN 5140 analyzer (EKF-Diagnostik, Barleben/Magdeburg, Germany).

### Quality controls of the metabolic systems

Several forms of quality controls of the metabolic systems were undertaken and are described in detail elsewhere (16–18). They will therefore only be briefly mentioned here. Since the SMS measured the oxygen uptake per minute (VO_2_) in the laboratory and the MMS measured it in the field, it was important to check the comparability of these two measurement systems. A high level of resemblance was noted in the VO_2_ range between 1 and 5 L · min^−^^1^ (R^2^ = 0.989) (18). The stability of SMS and MMS with time passing during the measurement series was established through checks with a metabolic calibrator (18). Furthermore, during 45 minutes of exercise in stationary field conditions we noted a stability in VO_2_ measurements of the MMS when a drying unit for the sample of the expired air was applied before it enters into the gas analyzing units of the MMS (17). Moreover, external wind was shown not to affect the VO_2_ measurements of the MMS (17). A number of other validity checks of the MMS were undertaken during the field studies, and included the effect of possible alterations in calibration factors. The combined effect of drifts in them amounted to −2.11 ± 2.77% (*n* = 15) for the whole period between the pre- and post-calibrations (18). Overall, the accumulated evidence from the quality control studies indicates that deviations in MMS, from the correct values, appear to be small, and of the magnitude of just some few per cent.

### Measurements

In the laboratory, the participants were tested at submaximal and maximal work rates on three different occasions; twice on an ergometer cycle followed by once on a treadmill while the metabolic variables were measured with the SMS. The reason for repeating the cycle ergometer test protocol was to familiarize the participants with the process, and to register if there was any learning effect between the first and second occasion (22). Metabolic measurements were then made during the participants' normal daily commutes using the MMS. Data from the second cycle ergometer test was used, except for in four cases where the field tests were delayed by 9–12 months due to technical problems with the MMS, which had to be solved and the equipment re-evaluated. A third cycle ergometer test in the laboratory was therefore deemed necessary to ensure valid comparisons between the laboratory and field tests. The mean time between the reference test on cycle ergometer in the laboratory, and the field test, was 23 ± 19 days.

Two trained investigators carried out the laboratory tests, each participant had the same investigator for each test. Three investigators carried out the field tests, with the same investigator in charge of the metabolic measurements. The participants were not able to drink during any of the tests because of the mask covering their mouths.

#### Laboratory tests

The participants were asked to follow standard procedures before each test occasion in the laboratory. These were: (1) not to engage in any vigorous exercise for 24 h beforehand, (2) not to cycle to the laboratory, (3) to refrain from eating, drinking, smoking and taking snuff for at least 1 h before arrival at the laboratory, (4) not to eat a large meal at least 3 h before the tests, (5) to avoid stress and (6) to cancel the test if they had fever, an infection or a cold. On arrival at the laboratory a check list was ticked off to determine if the participants had followed these standard procedures. They were then weighed and measured. A Polar heart rate watch and wear link were then placed on a wrist and around the chest, respectively. The participants thereafter rested quietly in a supine position for 10 minutes on a treatment table. Resting heart rate was determined from the average of 5 min from the 6^th^ to the 10^th^ min. Thereafter the resting blood pressure was measured while the participants remained lying in a supine position.

#### Cycle ergometer and treadmill exercise protocol

An evaluation of two test protocols on cycle ergometer was performed prior to the laboratory tests in order to find the most suitable one for reaching maximal oxygen uptake in normal healthy people, and to see if there were any differences between the first and second test occasions [cf. (21)]. These tests motivated the following protocol.

Participants cycled at three different work rates, 50, 100 and 150 watt (W) for the females, and 100, 150 and 200 W for the males. A cadence of 50 revolutions per minute (rpm) was chosen [(23), p. 19]. The participant cycled until steady state was reached at each work rate (~6 min). Thereafter the resistance was increased. If, after the second work rate, the subject's HR was higher than 150 beats · min^−1^, and their rated perceived exertion (RPE) exceeded 15 for legs and breathing, respectively [(24), p. 30], then the third work rate was increased to only 125 W for females and 175 W for males. The HR from the Polar watch was noted in the protocol after every minute, and RPE was noted at the end of each work rate.

Between the second and third work rates, the participant continued to cycle for 1 min at a self-chosen low cadence with a resistance of 5 Newton. The subject was then asked to resume the cadence of 50 rpm while the investigator slowly increased the work rate until, after 1 min, the third work rate was reached (resistance was increased to 50 W during the first 15 seconds, to 100 W the second 15 s and successively to the required work rate during the last 30 s). After the submaximal test was completed, the subject continued to cycle for 2 min at a self-chosen low cadence at 5 Newton.

During the maximal phase, the subjects cycled at a cadence of 80 rpm, since it is associated with the longest time to exhaustion in maximal efforts (25). For the first 3 min, the work rates were 60, 100, and 120 or 140 W for 1 min each. The latter alternatives depended on which third work rate the subjects had achieved during the submaximal work: 120 W if the third submaximal work rate had been 125 W or 175 W for females and males, respectively; 140 W if it had been 150 W or 200 W for females and males, respectively. The work rate increased thereafter by 20 W every 60 s. The test continued until exhaustion. HR was noted before each increase of the resistance, as well as at the moment when the participant terminated the test because of exhaustion.

The treadmill exercise protocol consisted of continuous level (0.0°) walking at the speeds 4, 5 and 6 km · h^−1^ until steady state values for HR was attained (normally 5 min). Immediately after completing the third walking load, the speed was increased to a comfortable level for running, after which the maximal phase was started. The maximal running test was performed through constant speed (9.0 ± 0.7 km · h^−1^) and successive increments of inclination. During the first minute of the test, the inclination was set to 0.0°, after 1 min it was increased to 1.0° and then by 0.5° every continued minute until voluntary exhaustion occurred and the test was terminated.

For all forms of submaximal work rates, paired HR and VO_2_ values during the last, of the two consecutive minutes, at steady state, have been used as averages for each workload. The values for the maximal tests in both ergometer cycling and treadmill running were calculated by averaging the highest consecutive values for VO_2_ and HR over 1 minute at maximal exercise. The same period was used for both VO_2_ and HR.

The Borg scale was used to assess the RPE [(24), p. 30]. The subjects were instructed on how to use the scale before commencing the tests and asked to point to a number on the scale that corresponded to their RPE for breathing and in their legs, respectively, before every increase of resistance during the submaximal test, and directly after the maximal test. To ensure that each subject achieved maximal exertion, at least two of the following three criteria were met by each subject: (1) a plateau in VO_2_ despite increasing power output (defined as a VO_2_ increment of < 150 mL · min^−1^), (2) an RER of ≥ 1.1, and (3) a rating of RPE of ≥ 17 (26–28).

#### Field measurements during walking commuting

All field measurements were undertaken between May and January, and the participants commuted either to or from their workplace, choosing themselves which time and direction was most convenient. Eighteen of the pedestrians were tested in the morning rush hours, and the remaining two were tested in the evening rush hours. The commuting walking took place in the inner urban and/or suburban areas of Greater Stockholm, Sweden, which are described below. The pedestrians were met at the designated address by one of the investigators who transported the equipment there by car. The investigator checked with the participant that the standard procedures before a measurement occasion, as described above in “Laboratory tests,” had been followed. The MMS (see preparation of MMS above) was placed in a specially designed backpack on the participant, and the gas sampling sensors were attached to the previously fitted mask. After confirmation that a recording was taking place, the measurement was started. HR was also measured using the Polar electro heart rate recorder. A GPS was placed in the backpack to track the route. This was used for comparisons with the routes drawn on maps by the pedestrians. A step counter (Digiwalker 200 SW, YAMAX, Japan), checked for validity, was placed in a belt at the hip, aligned vertically over the knee. Starting time of the walking trip was synchronized with the second investigator waiting at the destination. The participants were not able to drink during their walking commute because of the mask covering their mouths.

On arrival at the destination the pedestrians were met by the other investigator. The total trip time was noted, and the participant was asked to rate the overall RPE for both breathing and legs, respectively. The number of steps taken was registered, and within 2 min after arrival, blood samples for lactate analyses were taken. The participant then stated how many stops they made at traffic lights, as well as other stops, and marked them on maps with their routes. They were also asked to confirm whether that route had been taken the whole way, and if not, any deviation from the originally marked route was added in the map.

Oxygen uptake and other measures coupled to the walking were measured continuously and averaged for every 15 second period from the start to the end of the trip time, and then averaged for the whole commuting trip.

### Calculations of MET values and energy expenditure

1 MET is here defined as the oxygen uptake in mL · kg^−^^1^ · min^−^^1^ equal to the oxygen cost of sitting quietly, which is equivalent to 3.5 mL O_2_ · kg^−^^1^ · min^−^^1^ according to the data from Balke (29). The average MET value during each individual's walking commute was calculated as multiples of the participant's average VO_2_ during the whole commute, expressed as mL O_2_ · min^−^^1^, divided by the participant's body weight (kg) and divided by 3.5 mL O_2_ · min^−^^1^ (29). Our rationale for using the definition and values connected to “quietly sitting” is to be able to calculate the additional energy expenditure of the walking commuting compared to the most frequent sedentary position during waking hours, including sitting in cars or in public transit. The caloric coefficients for oxygen used in the energy expenditure calculations were based on non-protein respiratory quotients [(30), p. 104].

### Geographical area for the field tests

The pedestrians commuted in the inner urban and/or suburban – rural areas of Greater Stockholm, Sweden. Depending on if they walked in either one of these areas the routes were classified as “0” or “2,” and with “1” if the routes were partly in both of these areas. The boundary between these areas is described in Wahlgren et al. (31), and a detailed geographical description of them is given in Wahlgren and Schantz (32). For the evaluation of this study, it is relevant to know that the topography is generally rather flat, but gentle slopes of not more than about 10–15 meters height exist. Furthermore, as an indication of residential density of the suburban parts of our study area, we have chosen the southern and westerns suburbs of the Municipality of Stockholm as examples. In 2005 that density amounted to ~3,500 and 2,900 residents per km^2^, respectively, while in the inner urban area it amounted to 13 000 residents per km^2^ [(33), p. 95].

### Ambient conditions during the walking commuting

The average ambient conditions (temperature, relative humidity and wind speed) during the walking trips were obtained from the website: The Stockholm-Uppsala Air Quality Management Association (34). These are shown in Table 2. There were no significant sex differences in ambient conditions.

**Table 2:** Ambient conditions in walking commuting (mean and SD).

**Walking commuters**	**Temperature**	**Relative humidity**	**Wind speed**
	°**C**	**%**	**m** ·**s**^−1^
Men (*n* = 10)	**14.2** 8.4	**66.5** 19.5	**3.58** 1.07
Women (*n* = 10)	**15.5** 4.5	**64.1** 22.0	**3.61** 1.59

### Statistical analyses

Statistical analyses were performed using the Statistical Package for the Social Sciences (SPSS, 27.0, Chicago, IL, USA). Results are presented as means and standard deviation (SD) as well as 95% confidence interval (CI) unless otherwise stated. The significance level was set at *p* < 0.05.

Before any comparative analyses were performed, the normality of distribution was checked for all variables with Shapiro-Wilk test for males and females separately. Thereafter, all differences between the sexes were analyzed with the independent T-test. In cases where the data were not normally distributed, variables were also analyzed with the non-parametric test, Mann-Whitney U, for independent samples. Those variables with a significant sex difference, did not differ, however, in terms of significance between the T-test and the Mann-Whitney U test. All significant sex differences were calculated in both absolute and relative terms. These differences as well as effect size values for Hedges' correction are reported in Supplementary material 1. Finally, the one sample T-test was used to compare the ratio in maximal oxygen uptake between treadmill running and ergometer cycling vs. 1.0.

## Results

### Responses to maximal exercise in the laboratory

The maximal oxygen uptake was 11.0 ± 0.1% higher in treadmill running than in ergometer cycling (*p* < 0.05) (Tables 3, 4). Both these measures are therefore used as reference values to the oxygen uptakes during the walking commuting.

**Table 3:** Maximal values of heart rate, oxygen uptake, MET and rated perceived exertion in maximal ergometer cycling as well as the oxygen uptake of 1 MET (mean, SD and 95% CI).

**Walking commuters**	**Oxygen uptake of 1 MET**	**Maximal heart rate**	**Maximal oxygen uptake and MET**			**Maximal rated perceived exertion** **legs**	**Maximal rated perceived exertion breathing**
	**L** ·**min**^−^^1^	**beats** ·**min**^−^^1^	**L** ·**min**^−^^1^	**mL** ·**kg**^−^^1^ ·**min**^−^^1^	**MET**		
Men (*n* = 10)	**0.241** 0.036 (0.216–0.267)	**184** 8 (178–190)	**3.01** 0.46 (2.68–3.34)	**37.6** 5.6 (33.6–41.6)	**12.6** 1.8 (11.3–13.9)	**18.2** 1.0 (17.5–19.0)	**18.0** 1.0 (17.2–18.8)
Women (*n* = 10)	0.183^*^ 0.025 (0.165–0.201)	**175** 13 (166–184)	2.30^*^ 0.21 (2.16–2.45)	**38.7** 4.5 (35.5–41.8)	**12.7** 1.6 (11.6–13.9)	**19.0** 1.1 (18.2–19.8)	**18.9** 0.9 (18.3–19.5)

* Significant differences (p < 0.05) between men and women.

**Table 4:** Maximal values of heart rate, oxygen uptake, MET and rated perceived exertion in maximal treadmill running (mean, SD and 95% CI).

**Walking commuters**	**Maximal heart rate**	**Maximal oxygen uptake and MET**			**Maximal rated perceived exertion** **legs**	**Maximal rated perceived exertion breathing**
	**beats** ·**min**^−^^1^	**L** ·**min**^−^^1^	**mL** ·**kg**^−^^1^ ·**min**^−^^1^	**MET**		
Men (*n* = 10)	**185** 11 (177–193)	**3.31** 0.38 (3.04–3.58)	**41.7** 6.2 (37.3–46.2)	**13.9** 2.1 (12.4–15.4)	**17.3** 1.1 (16.5–18.1)	**17.4** 2.0 (16.0–18.8)
Women (*n* = 10)	**180** 10 (172–187)	2.56^*^ 0.27 (2.36–2.75)	**42.2** 5.3 (38.5–46.0)	**14.1** 1.8 (12.8–15.3)	**18.3** 1.3 (17.2–19.3)	**18.8** 1.0 (17.9–19.6)

* Significant differences (p < 0.05) between men and women. N = 8 in maximal rated perceived exertion for the women.

### Characteristics of the walking commutes

Characteristics of the walking commutes are listed in Table 5. Most variables did not differ between the sexes.

**Table 5:** Characteristics of trips, walking environments and stops for different reasons in walking commuting (mean, SD and 95% CI).

**Walking commuters**	**Duration**	**Distance**	**Speed**	**Trips per year**	**Walking environment**	**Stops for red lights**	**Other stops**
	**min**	**km**	**km** ·**h**^−^^1^				
Men (*n* = 10)	**25.6** 12.1 (17.0–34.3)	**2.39** 0.96 (1.71–3.07)	**5.71** 0.40 (5.42–5.99)	**364** 123 (276–452)	**0.20** 0.63 (−0.25–0.65)	**1.70** 1.49 (0.63–2.77)	**0.00** 0.00 (0.00–0.00)
Women (*n* = 10)	**20.9** 8.2 (15.0–26.8)	**2.05** 0.81 (1.48–2.63)	**5.87** 0.37 (5.61–6.14)	**405** 105 (330–480)	0.90^*^ 0.74 (0.37–1.43)	**1.20** 1.69 (−0.01–2.41)	**0.10** 0.32 (−0.13–0.33)

* Significant differences (p < 0.05) between men and women. Walking environment: 0, inner urban; 1, inner urban – suburban; 2, suburban. For more information about the walking environments, see Methods under the heading “Geographical area for the field tests”.

### Characteristics of the walking steps

Characteristics of the walking steps are listed in Table 6. The step frequency differed between the sexes.

**Table 6:** Characteristics of steps and step frequency in walking commuting (mean, SD and 95% CI).

**Walking commuters**	**Number of steps**	**Step length**	**Step frequency**		**Step length relative to body height**
		**m**	**steps** ·**m**^−^^1^	**steps** ·**min**^−^^1^	**m** ·**m**^−^^1^
Men (*n* = 10)	**2 945** 1 308 (2 009–3 881)	**0.820** 0.053 (0.782–0.859)	**1.22** 0.08 (1.17–1.28)	**116** 4 (113–119)	**0.458** 0.033 (0.435–0.482)
Women (*n* = 10)	**2 602** 1 064 (1 841–3 364)	**0.795** 0.047 (0.761–0.829)	**1.26** 0.08 (1.21–1.32)	123^*^ 5 (120–127)	**0.472** 0.027 (0.453–0.491)

* Significant differences (p < 0.05) between men and women.

### Oxygen uptake and MET values in walking commuting per unit of time

The absolute levels of oxygen uptake per minute and kg body weight, the MET values, and the relative intensity of walking commuting in per cent of oxygen uptake did not differ between the sexes (Table 7).

**Table 7:** Oxygen uptake per minute (total and minus 1 MET), per cent of maximal oxygen uptake, and MET in walking commuting (mean, SD and 95% CI).

**Walking commuters**	**Oxygen uptake** **per minute**		**Per cent of maximal oxygen uptake based on ergometer cycling**	**Per cent of maximal oxygen uptake based on treadmill running**	**MET**	**Oxygen uptake – 1 MET per minute**	
	**L** ·**min**^−^^1^	**mL** ·**kg**^−^^1^ ·**min**^−^^1^	**%**	**%**		**(L - L)** ·**min**^−^^1^	**(mL - mL)** ·**kg**^−^^1^ ·**min**^−^^1^
Men (*n* = 10)	**1.50** 0.32 (1.27–1.73)	**18.6** 3.1 (16.4–20.9)	**49.8** 7.1 (44.7–54.9)	**45.5** 10.0 (38.3–52.7)	**6.22** 1.03 (5.48–6.95)	**1.26** 0.30 (1.04–1.47)	**15.6** 3.1 (13.4–17.9)
Women (*n* = 10)	1.18^*^ 0.16 (1.07–1.30)	**19.5** 2.6 (17.6–21.4)	**51.5** 6.7 (46.7–56.3)	**46.6** 6.5 (41.9–51.2)	**6.51** 0.87 (5.88–7.13)	1.00^*^ 0.15 (0.89–1.11)	**16.5** 2.6 (14.6–18.4)

* Significant differences (p < 0.05) between men and women.

### Energy expenditure in walking commuting per unit of time

The absolute levels of energy expenditure in walking commuting per minute and kg body weight did not differ between the sexes. The same applies to the levels of energy expenditure minus 1 MET, i.e., the added energy expenditure by the commuter walking *per se* (Table 8).

**Table 8:** Respiratory exchange ratio (VCO_2_ · VO${}_{2}^{-}$^1^), caloric coefficient (cal · VO${}_{2}^{-}$^1^), energy expenditure (total and minus 1 MET) in walking commuting (mean, SD and 95% CI).

**Walking commuters**	**Respiratory exchange ratio**	**Caloric coefficient**	**Energy expenditure per minute**		**Energy expenditure – 1 MET per minute**	
	**L** ·**L**^−^^1^	**kcal** ·**(L** ·**min**^−1^**)**^−1^	**kcal** ·**min**^−^^1^	**kcal** ·**kg**^−^^1^ ·**min**^−^^1^	**(kcal - kcal)** ·**min**^−^^1^	**(kcal - kcal)** ·**kg**^−^^1^ ·**min**^−^^1^
Men (*n* = 10)	**0.872** 0.035 (0.846–0.897)	**4.89** 0.04 (4.86–4.92)	**7.33** 1.58 (6.19–8.46)	**0.0911** 0.0150 (0.0804–0.1019)	**6.15** 1.48 (5.09–7.21)	**0.0765** 0.0150 (0.0657–0.0872)
Women (*n* = 10)	**0.842** 0.046 (0.809–0.874)	**4.85** 0.06 (4.81–4.89)	5.74^*^ 0.80 (5.17–6.32)	**0.0947** 0.0124 (0.0858–0.1036)	4.85^*^ 0.75 (4.32–5.38)	**0.0801** 0.0125 (0.0712–0.0890)

* Significant differences (*p* < 0.05) between men and women.

### Heart rate, per cent of maximal heart rate, per cent of heart rate reserve, ventilation, ventilatory equivalent, blood lactate and rated perceived exertion in walking commuting

The absolute and relative levels of heart rate and blood lactate did not differ between the sexes (Table 9).

**Table 9:** Heart rate, per cent of maximal heart rate (%HRmax), per cent of heart rate reserve (%HRR), ventilation, ventilatory equivalent (VE · VO_2_^−^^1^), blood lactate and rated perceived exertion (RPE) in walking commuting (mean, SD and 95% CI).

**Walking commuters**	**Heart rate**	**Per cent of maximal heart rate**	**Per cent of heart rate reserve**	**Ventilation (BTPS)**	**Ventilatory equivalent**	**Breathing frequency**	**Tidal volume**	**Blood** **lactate**	**Rated perceived exertion** **leg**	**Rated perceived exertion breathing**
	**beats** ·**min**^−^^1^	**%**	**%**	**L** ·**min**^−^^1^	**L** ·**L**^−^^1^	**breaths** ·**min**^−^^1^	**L**	**mmol** ·**L**^−^^1^		
Men (*n* = 10)	**113** 16 (102–124)	**61.3** 8.1 (55.6–67.1)	**35.5** 12.3 (26.8–44.3)	**39.1** 10.3 (31.8–46.5)	**25.9** 2.6 (24.1–27.8)	**23.4** 4.4 (20.3–26.5)	**1.67** 0.29 (1.46–1.88)	**1.02** 0.48 (0.67–1.36)	**10.7** 1.3 (9.8–11.6)	**11.2** 1.8 (9.9–12.5)
Women (*n* = 10)	**111** 10 (104–118)	**63.7** 6.1 (59.4–68.1)	**44.2** 8.2 (38.3 – 50.1)	31.2^*^ 5.2 (27.5–34.9)	**26.4** 3.2 (24.1–28.7)	**25.0** 3.9 (22.2–27.8)	1.26^*^ 0.22 (1.10–1.42)	**1.24** 0.46 (0.88–1.59)	8.70^*^ 1.57 (7.58–9.82)	9.40^*^ 1.58 (8.27–10.53)

* Significant differences (p < 0.05) between men and women. %HRmax and %HRR are calculated on the basis of HRmax in ergometer cycling. The corresponding values in relation to HRmax in treadmill running were: 61.2 ± 8.3 (55.2–67.1) for the men, and 61.9 ± 5.1 (58.3–65.5) for the women (%HRmax). %HRR were 35.4 ± 12.3 (26.6–44.2) for the men, and 42.2 ± 7.4 (36.9–47.5) for the women. The blood lactate values for the women are based on 9 participants.

### Oxygen uptake and energy expenditure in walking commuting per unit of distance

The absolute levels of oxygen uptake and energy expenditure in walking commuting per km did not differ between the sexes, when dividing them with the body weight. The same applies to the levels of oxygen uptake and energy expenditure minus 1 MET (Tables 10, 11).

**Table 10:** Oxygen uptake (total and minus 1 MET) per distance in walking commuting (mean, SD and 95% CI).

**Walking commuters**	**Oxygen uptake** **per distance**		**Oxygen uptake –** **1 MET per distance**	
	**L** ·**km**^−^^1^	**mL** ·**kg**^−^^1^ ·**km**^−^^1^	**(L - L)** ·**km**^−^^1^	**(mL - mL)** ·**kg**^−^^1^ ·**km**^−^^1^
Men (*n* = 10)	**15.9** 4.0 (13.0–18.7)	**197** 32 (173–220)	**13.3** 3.6 (10.7–15.9)	**165** 32 (142–188)
Women (*n* = 10)	12.1^*^ 1.3 (11.1–13.0)	**199** 21 (184–215)	10.2^*^ 1.2 (9.3–11.1)	**168** 22 (153–184)

* Significant differences (p < 0.05) between men and women.

**Table 11:** Energy expenditure (total and minus 1 MET) per distance in walking commuting (mean, SD and 95% CI).

**Walking commuters**	**Energy expenditure per distance**		**Energy expenditure –** **1 MET per distance**	
	**kcal** ·**km**^−^^1^	**kcal** ·**kg**^−^^1^ ·**km**^−^^1^	**(kcal - kcal)** ·**km**^−^^1^	**(kcal - kcal)** ·**kg**^−^^1^ ·**km**^−^^1^
Men (*n* = 10)	**77.7** 19.4 (63.7–91.6)	**0.960** 0.157 (0.848–1.072)	**65.1** 17.5 (52.6–77.7)	**0.805** 0.155 (0.695–0.916)
Women (*n* = 10)	58.6^*^ 6.6 (53.8–63.3)	**0.966** 0.101 (0.894–1.038)	49.5^*^ 6.0 (45.2–53.8)	**0.817** 0.103 (0.744–0.891)

* Significant differences (p < 0.05) between men and women.

### Oxygen uptake and energy expenditure per step of walking commuting

Levels of oxygen uptake and energy expenditure per step did not differ between males and females when taking into account the body weight (Table 12).

**Table 12:** Characteristics of the oxygen uptake and energy expenditure per walking commuting step (total and minus 1 MET) (mean, SD and 95% CI).

**Walking commuters**	**Oxygen uptake**				**Energy expenditure**			
	**Oxygen uptake per step**	**Oxygen uptake per step and body weight**	**Oxygen uptake – 1 MET per step**	**Oxygen uptake – 1 MET per step and body weight**	**Calories per step**	**Calories per step and body weight**	**Calories - 1 MET per step**	**Calories - 1 MET per step and body weight**
	**mL** ·**step**^−1^	**mL** ·**step**^−1^ ·**kg** ^−1^	**(mL – mL)** ·**step**^−1^	**(mL – mL)** ·**step**^−1^ ·**kg** ^−1^	**cal** ·**step**^−1^	**cal** ·**step**^−1^ ·**kg** ^−1^	**(cal – cal)** ·**step**^−1^	**(cal – cal)** ·**step**^−1^ ·**kg** ^−1^
Men (*n* = 10)	**13.0** 3.0 (10.8–15.1)	**0.161** 0.028 (0.141–0.181)	**10.9** 2.8 (8.9–12.9)	**0.135** 0.027 (0.116–0.155)	**63.4** 14.7 (52.9–73.9)	**0.787** 0.134 (0.691–0.883)	**53.2** 13.6 (43.5–62.9)	**0.661** 0.133 (0.565–0.756)
Women (*n* = 10)	9.60^*^ 1.25 (8.71–10.50)	**0.158** 0.020 (0.144–0.173)	8.11^*^ 1.16 (7.28–8.94)	**0.134** 0.020 (0.120–0.148)	46.6^*^ 6.2 (42.2–51.0)	**0.768** 0.093 (0.702–0.835)	39.4^*^ 5.7 (35.3–43.4)	**0.650** 0.094 (0.583–0.717)

* Significant differences (p < 0.05) between men and women.

### MET-hours and energy expenditure in walking commuting per week, and the accumulated energy expenditure and distance over a year

The estimated number of MET-hours and energy expenditure per week in the month of May, with a trip frequency per week of 8.8 ± 2.2 for men and 8.7 ± 2.2 for women, as well as values for the accumulated energy expenditure and distance walked over a year are given in Table 13. The energy expenditure for the mean trip length of 2.39 ± 0.96 km for men was 194 ± 116 kcal. For women, the corresponding values were 2.05 ± 0.81 km and 122 ± 53 kcal. No differences were noted between the sexes.

**Table 13:** Trip frequency per week, estimated MET-minutes, MET-hours and energy expenditure per week in May as well as the accumulated distance walked and energy consumed in walking commuting over a year (mean, SD and 95 % CI).

**Walking commuters**	**Frequency of trips per week in May**	**MET-minutes per week in May**	**MET-hours per week in May**	**Energy expenditure per week in May**	**Total distance walked per year**	**Total energy expenditure per year**	**Total energy expenditure – 1 MET per year**
	**trips** ·**week**^−^^1^	**MET-minutes** ·**week**^−^^1^	**MET-hours** ·**week**^−^^1^	**kcal** ·**week**^−^^1^	**km** ·**year**^−^^1^	**kcal** ·**year**^−^^1^	**(kcal – kcal)** ·**year**^−^^1^
Men (*n =* 10)	**8.80** 2.15 (7.26–10.34)	**1 307** 516 (937–1 676)	**21.8** 8.6 (15.6–27.9)	**1 566** 738 (1 038–2 094)	**792** 249 (614–970)	**63 139** 31 988 (40 256–86 022)	**53 236** 28 590 (32 784–73 688)
Women (*n* = 10)	**8.70** 2.16 (7.15–10.25)	**1 187** 591 (765–1 610)	**19.8** 9.8 (12.7–26.8)	**1 036** 491 (684–1 387)	**807** 335 (568–1 047)	**47 632** 21 285 (32 405–62 859)	**40 321** 18 413 (27 149–53 493)

## Discussion

This study examines essential movement features of regular walking commuting using validated methodology for all variables except for self-reported trip frequency. Physiological and behavioral data are combined to form a broad base to elucidate walking commuting in relation to previous studies on morbidity and premature mortality, aerobic fitness and control of body weight. Data on oxygen uptake and energy expenditure are presented, and also depicted with and without the influence of 1 MET, so that the exercise induced changes stand out compared to sitting still.

An important finding is that walking in a field setting demanded about 30% more energy per km than level walking. Another finding is that males and females walk at a similar mean aerobic intensity of 46% of their VO_2_max, and with an energy utilization of 0.96 kcal per kg body weight and km. When combining trip frequency, duration and MET values, high values of MET-hours per week in the month of May (22 for males and 20 for females) were noted. These levels of walking are sustained over the year (12) and result in about 385 trips and 800 km of commuter walking per year for both sexes.

We will discuss these findings separately, and in relation to exercise capacity, weight control and other health related variables. We will also compare commuter walking and cycling, which is of interest in itself, as well as given the background that two recent studies indicate they may have different effects on health outcomes (13, 14).

### Walking characteristics

Walking characteristics can be divided into several variables such as duration of episodes of walking, number and length of steps, cadence and speed.

When the seminal report “Physical Activity and Health” was launched in 1996 by the U.S. Department of Health and Human Services (1), a minimum intensity of brisk walking (3–4 mph/4.8–6.4 km · h^−1^) was recommended in order to gain health effects. The current mean velocities (5.7; 5.9 km · h^−1^) meet those recommendations.

Several studies have tried to establish cadences for different age groups corresponding to moderate exercise intensity. A recent study by McAvoy et al. (11), using RPE, %HRR, and %HRmax to establish a moderate exercise intensity on the treadmill, suggests that a cadence between 117.7 and 122.7 is relevant for the age group 40-50 years. In relation to their findings, it is worth noting that in the present study, mean cadences of 116 and 123, respectively, for normal weight males and females in the same age group, were associated with being at the lower limit of the moderate walking intensity when related to % of VO_2_max (see below).

Whether cadence is a good indicator of exercise intensity can, however, be questioned. Recent findings based on level walking on the treadmill demonstrate large variations at given cadences in both relative exercise intensities (11) and in absolute oxygen uptake per kg body weight and minute (35). To further characterizations of walking, it would be of clear value if cadence is coupled to step length so that speed of walking can be established.

Several studies have aimed at characterizing level walking outdoors at different paces [cf. (36)]. For the category “usual” pace we have compared their findings with the present (Table 14). While cadences are almost the same in the two studies, we note divergences in step length, speed, and oxygen uptake. This points again to the value of including cadence, step length and speed when characterizing walking.

**Table 14:** A comparison between mean data from Murtagh et al. (36) and the present study (37).

**Study**	**Speed**		**Cadence**	**Step length**	**Oxygen uptake per body weight and minute**
	**km** ·**h**^−^^1^	**m** ·**s**^−^^1^	**steps** ·**min**^−^^1^	**m**	**mL** ·**kg**^−^^1^ ·**min**^−^^1^
Murtagh et al. (36)	4.72	1.31	117	0.674	12.0
Schantz et al. (37); men	5.71	1.59	116	0.820	18.6
Schantz et al. (37); women	5.87	1.63	123	0.795	19.5
Schantz et al. (37); mean for men and women	5.79	1.61	120	0.808	19.1
Ratio Schantz/Murtagh	1.23	1.23	1.03	1.20	1.59

In Supplementary material 2 we present unpublished data of level treadmill walking at different speeds by males and females. They support the values noted by Murtagh et al. (36) regarding mL of oxygen uptake per kg body weight and minute (see Table 14 and Supplementary material 2).

Based on Table 14 and Supplementary material 2 it can be calculated that the levels of oxygen uptake per kg body weight and minute for a certain speed during the studied commuter walking are 27–33% higher than at level walking for the pedestrians and other groups of participants. This also means that the energy cost per km is about 30% higher during commuter walking than at level walking. We interpret this as being due to primarily vertical work components during the commutes because of slopes. Uphill walking on a 5% slope at speeds of the present commuting pedestrians (5.8 km · h^−^^1^) increases the energy demands by about 50% compared to level walking (38), while walking downhill, on a corresponding slope, may decrease the energy consumption in comparison with level walking [cf. (39)].

Such ingredients in the topography of the landscape also create longer distances than those measured on the maps. This is due to longer hypotenuses introduced by slopes, in comparison with the horizontal catheti. Since our speed calculations are based solely on the map measured horizontal distance, the actual speeds of the walking commuters will be slightly higher than stated here.

We therefore recommend that future studies of naturalistic walking include an index for how oxygen uptake and energy demands [cf. (39)] as well as distances change with different relative components in terms of horizontal, up- and downhill walking. Data on the latter should be rather easy to obtain through tracing walking paths with global positioning systems and combining the data with topography analyses by geographic information systems.

The minimal level of walking steps for transport per day, to gain health effects, is stated as 3,000 by Tudor-Locke et al. (40). The present walking commuters reached mean levels of 5,200 and 5,900 steps per day in two trips. When combining these steps with the associated levels of oxygen uptake, this walking behavior comes closer to the optimal levels of physical activity recommendations in terms of number of steps per day, associating it with considerable health effects [cf. (4–6)].

### Intensity category

Both relative and absolute exercise intensities are stated by The American College of Sport Medicine (ACSM) when they relate to sustained health in adults (9). Their exercise intensity categories are termed as “very light,” “light,” “moderate,” “vigorous,” and “near maximal to maximal.” Our study indicates that commuter walking is in the lower range of the moderate intensity category according to ACSM (46–63% of VO_2_max), which is, overall, supported by the values for %HRmax and %HRR. The present RPE values for breathing (9.4–11.2) suggest that the relative intensity is light (9). However, this can be a falsely low categorization, since lower RPE values can be anticipated for a given work rate when moving through a landscape rather than being physically active indoors (41).

On the other hand, when considering the categorization in absolute intensities in relation to the age of the participants, their MET values during walking (6.2–6.5) are in the lower section of the vigorous category according to ACSM. Blood lactate levels, although not part of the ACSM-measures, point to walking being characterized by a moderate intensity level as they were well below the 4 mmol levels [(42), p. 432].

The ventilatory equivalent, i.e. the ventilation per minute divided by the oxygen uptake per minute, was ~26 for both sexes, which indicates moderate relative intensities [cf. (42), p. 228, (43)]. Taken altogether, it is reasonable to conclude that commuter walking creates demands in the lower part of the moderate relative intensity.

It is important to discuss the external validity of these findings. Active commuters who sometimes cycle and sometimes walk (double mode commuting), generally have longer walking distances and durations, but the same walking velocities compared to single mode pedestrians, i.e., those that only walk to work (12). The present single mode pedestrians' walking velocities, ages and BMI are of the same order of magnitude as for double mode commuters [cf. (12)], which suggests that the results of this study are representative for larger groups of walking commuters.

### MET values

Various forms of human movements are often assigned MET values in epidemiological studies. Most often they are based on Ainsworth's compendium from 1993 (44). In the latest version (45), the MET value 4 is stated for walking to work, whereas our measurements indicate average levels of 6.2–6.5. If focusing on the speed of walking instead, our values of 5.7–5.9 km per hour should, according to the compendium, correspond to about 4.3 MET when walking on a level and firm surface, and 5.3 MET when walking up a 1–5% grade hill. Still, it is clear that the presently measured values indicate sligthly higher MET levels than the 2011 compendium.

Moreover, according to Tudor-Locke et al. (46) a MET value of 6 (vigorous intensity) is associated with a step frequency of ~120–129 steps per minute. Thus, the present frequencies used in walking commuting (116 and 123 steps · min^−1^), further point to the actual MET values being higher than 4 MET.

### Energy expenditure and MET-hours

ACSM states that most adults should, in order to attain sustained health, participate in moderate intensity exercise either separately, or combined with exercise of vigorous intensity, with the aim of reaching a total energy expenditure of ≥8.33–16.7 MET-hours per week (9). If calculating an average range between minimal and optimal levels of physical activity according to WHO's guidelines from 2020, it amounts to 11.25–22.50 MET-hours per week (10).

During a typical week in May, the females and males in this study attained about 20 and 22 MET-hours per week, respectively. The walking commuting behaviors over the year are very stable in terms of trip frequency (12). Thereby, activity levels through walking to work during the year are close or above the optimal ACSM and WHO levels.

Let us exemplify potential health effects with two other positionings. In the Nurses' Health Study the highest quintile of leisure walking amounted to 22 MET-hours per week, and it was associated with the maximal reduction (42%) of incidence of type II diabetes (47). In the Harvard Alumni Study on males, leisure time physical activity with a weekly energy turnover of 1,500-1,999 kcal corresponded to a 37% decrease in all-cause mortality (48). These levels of energy demands were reached by the presently studied male pedestrians over the year, with the exception of the predominant vacation month of July (12).

### Ventilation

Ventilation levels during walking and cycling are the focus of several studies since negative impacts can be induced by inhaling polluted air [cf. (49, 50)]. The present version, from 2017, of the WHO tool for assessing health impacts of walking (HEAT) (51) incorporates the negative effects of air pollution. In this tool, the ventilation level for walking is set to 1.37 m^3^ per hour, whereas our results point to means of 2.35 m^3^ per hour for males, and 1.87 for females, with lower values for the 95% confidence intervals that are clearly above 1.37 m^3^ per hour. Thus, possible negative health effects when walking appear to be underestimated in the present version of the WHO HEAT tool. The same divergence in values was noted in commuter cyclists (19). These discrepancies need to be elucidated in future studies with validated measurements of ventilation, and reasonable movement speeds [cf. (50)].

### Maximal aerobic capacity

About 5–10% higher levels of maximal oxygen uptake can often be noted if maximal exercise is undertaken on a treadmill instead of a cycle ergometer [cf. (42), p. 335]. Such a difference was also seen in the present pedestrians. A consequence of this is that absolute levels of submaximal oxygen uptake, when defined as a percentage of maximal oxygen uptake, will differ depending on the mode for the maximal exercise. Thereby, results between studies will differ merely due to the different reference points. We have therefore presented data from both these types of maximal measurements and described the relative oxygen uptake in relation to VO_2_max attained in treadmill running and ergometer cycling, respectively. When stating that, for both sexes, the walking intensity was about 46% of the maximal oxygen uptake (Table 7), we have made use of the highest attained levels of maximal oxygen uptake that were coupled to the tests on treadmill.

Notably, there are two dimensions coupled to maximal aerobic capacity; (i) the maximal uptake of oxygen, and (ii) the capacity to make use of larger amounts of it during sustained exercise [(8), p. 421]. The combination of these two measures will limit the capacity for a maximal oxygen dependent physical work.

It appears that there is a relation between maximal oxygen uptake and health outcomes (52–55). However, does this stand for a relationship between energy expenditure in physical activity and health, or is there possibly an independent effect of maximal oxygen uptake? To our knowledge, this remains to be elucidated.

Increases in maximal oxygen uptake are generally viewed as depending on both the training status and the character of the physical exercise in terms of intensity, duration, and frequency. Each of the three latter aspects can modify the effect level. Exercise intensities from 50% of VO_2_max and above induce increased maximal aerobic capacity in untrained individuals [cf. (56)], whereas 50% of VO_2_max is not sufficient in somewhat aerobically trained persons, even if exercise durations amount to 6 h a day, 6 days per week for a period of 8 weeks (57, 58). However, this exercise volume can still lead to a higher relative utilization of the maximal oxygen uptake during prolonged exercise [(57), p. 17]. Thus, these two principally different outcomes of aerobic exercise can adapt differently.

Physical exercise seems to be able to increase VO_2_max in practically all non-trained individuals if only the duration, intensity and frequency are sufficient (59). Given that, will the pedestrians' mean intensity of 46% of VO_2_max, durations of 21–26 min, and weekly trip frequency of 9, lead to increased levels of VO_2_max? That it might be so is indicated by the estimated VO_2_max levels in our samples being about 17% higher than of the general population at the same age (60). But, these differences, or part of them, can also be due to selection factors.

Evidence that higher VO_2_max levels are at least partly due to training effects of the commuter walking comes from the ACSM position that exercise HR in the ranges of 60–90% of HRmax is effective for aerobic fitness training (61). The average levels for the pedestrians were about 62% of HRmax. However, in the type of training studies that has formed our understanding of effects of aerobic training, the intensities used are normally constant and tightly controlled, whereas they fluctuate during normal commuter walking. This opens up questions concerning the external validity of results from studies based on constant vs. fluctuating intensities. Indeed, this appears to be an important area for future studies.

As far as we know, there is only one longitudinal study on the effects of commuter walking in adults on absolute maximal aerobic capacity. It was based on healthy participants who did not exercise regularly and vigorously during their leisure time. They did not show an increase in VO_2_max (62). However, this could be due to the modest levels of trip frequency and/or duration used, as well as the length of the intervention. In terms of training effects on maximal work capacity (which also includes making use of the anaerobic capacity), responses follow a dose-response relationship, where initial fitness levels have been shown to be important in studies of cycle commuting (63). We are not aware of any such studies on walking.

### Weight control and supply of nutrients

To diminish obesity, it is known that some physicians ordinated walking already during the late 18^th^ century [(64), p. 67–71]. From the evolvement of the industrial era, the handling of overweight has attracted a growing and more widespread concern (65). Indeed, the epidemic of increasing body weight during the past three decades [e.g., (66)] has led to great interest into how physical activity behaviors can rectify this development. Another aspect of the value of physical activity that is less known, is that a higher energy turnover facilitates the intake of sufficient levels of essential nutrients [(8), p. 574–575. (67)].

Based on the literature, it is reasonable to conclude that active commuting can diminish overweight and support weight control. A cross-sectional analysis of UK Biobank data noted lower BMI and percentage of body fat in active commuters compared to both passive and public transport commuters (68). Furthermore, another cross-sectional study noted a relationship between lower amounts of visceral adipose tissue and active commuting (69). In line with this, the present walking commuters had mean BMI values of 21.5 for the females and 25.0 for the males.

The data on energy demands of walking commuting in this study, is, in this respect, valuable for different interpretations. With an energy amount of 6,000–7,000 kcal per kg of fat tissue [(42), p. 485], the energy expenditure of the male and female pedestrians amounted to around 8 and 6 kg of fat tissue per year, respectively, after deducting 1 MET from sitting still. According to Ross and Janssen (70), 0.10–0.15 kg body weight losses per week could be expected with the stated energy expenditures in walking commuting, and for at least up to 4 months. Thereafter, a weight loss through physical activity seems to diminish, and to be more difficult to achieve (70). The advantage of the weekly energy expenditures through walking commuting of around 1,000 kcal in females, and 1,600 kcal in males, should, however, also be seen in relation to converting low energy consumers (1,500–2,000 kcal · day^−^^1^) to high energy consumers (2,500–3,000 kcal · day^−^^1^). In this way, walking can facilitate reaching adequate levels of essential nutrients [(8), p. 574–575, (67)].

### Energy expenditure per distance

It is interesting to note that the energy consumption per walking distance is the same for both sexes (0.96 kcal · kg^−^^1^ · km^−^^1^). These values are about 15–30% higher than previously noted in level walking at the corresponding speeds [cf. (38); Supplementary material 2]. This can primarily be explained by the fact that there are vertical components included in the horizontal distances measured during the walking commuting. It is also interesting to note that the walking speeds used are effective in relation to the energy cost of walking per km, whereas if the pedestrians had increased their speeds, their energy costs would increase relatively more than the walking speeds [cf. (38); Supplementary material 2].

### Walking or cycling commuting – what should be recommended? Reflections on a comparison of relative and absolute intensities, speeds and energy demands

Our previous study focusing on cycle commuting (19), with many of the same variables as in this study, prompts a comparison with the present data on walking. For that purpose, sex neutral data has been created, and a body weight of 70 kg has been used to calculate oxygen demands and energy turnovers per unit of time and distance (Table 15).

**Table 15:** A sex and body weight neutral comparison between walking and cycling for commuting purposes.

	**Age**	**Body weight**	**Speed**	**Time per distance**	**Per cent of maximal heart rate**	**Per cent of maximal oxygen uptake**	**MET**	**Oxygen uptake per time and distance**		**Energy expenditure per time and distance**		**Rated perceived exertion** **leg**	**Rated perceived exertion breathing**
	**years**	**kg**	**km** ·**h**^−^^1^	**min** ·**km**^−^^1^	**%**	**%**		**L** ·**min**^−^^1^	**L** ·**km**^−^^1^	**kcal** ·**min**^−^^1^	**kcal** ·**km**^−^^1^		
**Walking**	45.2	70	5.8	10.3	62.5	46.0	6.36	1.33	13.7	6.50	67.0	9.70	10.3
**Cycling**	43.8	70	18.4	3.3	78.0	64.7	8.09	1.98	6.54	9.80	32.3	11.4	12.6
**Ratio walking** **/cycling**	1.03	1.0	0.32	3.12	0.80	0.71	0.79	0.67	2.09	0.66	2.07	0.85	0.82

Calculations are based on average data from the present study of walking and of cycling from Schantz et al. (19). The maximal values for heart rate and oxygen uptake are based on the maximal ergometer cycling test. Rated perceived exertion can be affected by duration of exercise (71, 72). In this case, the mean durations of walking (23.2 min) and cycling (26.2 min) were almost the same.

This comparison indicates that both the relative (%HRmax, %VO_2_max and RPE) and the absolute measures of exercise intensity (MET and VO_2_) are higher during cycling than walking commuting. This is in line with Oja et al. (62). Still, given that the relative intensity is at least moderate in both walking and cycling commuting, effects related to both aerobic fitness and prevention of diseases are seen as possible to attain (see the previous discussion).

In our minds, the measures “energy turnover” and “MET-hours per week” coupled to physical activity are, at present, the only secure, if measured correctly, and objectively identifiable measures of physical activity outcomes in relation to premature mortality [e.g., (48)] and disease prevention [e.g., (47)]. Differences in health effects can thus be expected when walking and cycling a given distance or duration for commuting purposes (cf. Table 15). From a perspective of energy turnover, a given distance favors walking, whereas a given duration favors cycling. But will any difference exist if these modalities are compared during isocaloric circumstances or equal MET-hours per week?

Two studies have recently indicated differences in health effects with walking and cycling (13, 14). However, their findings might be due to not having taken differences in energy turnover and MET-hours into account, nor possible differences in durations and trip frequencies per week over the year.

These issues deserve to be studied in more depth than previously undertaken, and with more developed methods. Indeed, there is still a very limited basis for understanding which the crucial components of physical activity are in order to evoke preventive medical effects in relation to the rather great number of various diseases that physical activity appears to be able to affect [cf. (73)]. Furthermore, each of these effects might be induced along various biological pathways. Therefore, to sort out if there are any differences in health effects between walking and cycling commuting, it would be valuable to know more about the scope of exercise intensities, and the character of intensity undulations.

Presently, these kinds of data appear to be lacking. The fact that such exercise characteristics can be important is indicated by MacInnis et al. (74), who showed that continuous, as compared to intermittent exercise, but with a constant overall workload, evoked differences in the adaptive exercise response in mitochondrion. This emphasizes the importance of describing free-living exercise with respect to its potentially dynamic character. This demands novel ways of describing the metabolism that it evokes. Mean values during exercise need to be supplemented with data on the dynamic variability. Hopefully such data and study protocols can in future increase our understanding of relationships between free-living physical activity and its enigmatic diverse preventive effects on diseases [cf. (73)].

### Strengths and limitations

A strength of this study is that it is based on meticulous evaluations of measurements with metabolic systems in both laboratory and field conditions (16–18) (for more detailed comments, see below). Likewise, we measured route distances with a criterion method (15), walking durations were timed, body weights were measured, and the number of steps during the walking trip were counted with a valid step counter. The participants were representative for single mode pedestrians in a greater study sample [cf. (12)]. However, limitations were the small number of participants. In addition, the self-reported frequency of trips was not validated.

It was of crucial importance that the metabolic measuring systems used were valid throughout the data collection period. This included both the laboratory tests and prolonged field tests, as well as varying ambient conditions and relevant levels of metabolism. Considerable efforts were undertaken to ensure this (16–18).

The respiratory exchange ratio was the only variable in which a difference of some percent was noted between the MMS and the criterion method (16). It had a negligible effect on the caloric coefficient, and was within the uncertainty of a few percent in the overall levels of metabolism during commuter walking, as previously described (18).

Characteristics and behavior of the participants are other parts of the validity dimension. The present participants were habitual commuter pedestrians, who were chosen from a larger sample of individuals. Their characteristics were close to the larger sample's mean ages and median values for walking distances and speeds [cf. (12)]. In addition, males and females walked in very similar landscapes and ambient conditions, including wind speed (cf. Table 2).

Regarding external validity, how was the landscape topography in which the walking commutes took place? In general, it was quite flat, but there were smaller hills of up to about 10–15 meters height (see Methods). Thus, walking routes include varying proportions of horizontal and vertical components. When combined, they constitute individual variations in overall workloads for a given horizontal distance. And this can obviously also vary between different route landscapes. We therefore recommend that future studies of walking exercise in the field describe the topography of the landscape used in terms of accumulated horizontal and vertical distances. It would be ideal if an index could be developed for how oxygen uptake and walking speed change with different relative ingredients of horizontal, up- and downhill components during walking. Data on this should, as stated before, be rather easy to obtain through tracing walking paths with global positioning systems and combining the data with topography analyses by geographic information systems.

A final comment relates to the MET values for the pedestrians in relation to the averages in the normal population. Since the pedestrians appear to have about 17% higher levels of VO_2_max than the population, correspondingly lower MET values can be expected in the population.

## Conclusion

Overall, the present study stands for a novel basis for interpreting walking commuting in relation to various health outcomes. It concludes that walking commuting is characterized by equivalent relative aerobic intensities for male and female pedestrians. Corresponding similarities apply to energy demands for a given walking distance when differences in body weight are considered. However, given that the landscape walked in is not flat, we noted about 30% higher energy demands per km in comparison to level walking on a treadmill. In average terms, the walking commuting represents the lower part of the moderate relative intensity domain. The ventilation during these commutes was higher than the levels commonly used in studies focusing on possible negative effects of walking and air pollution. The combined levels of oxygen uptake, trip duration and frequency lead to high and sustained levels of weekly MET-hours over the year, as well as high levels of accumulated energy expenditure during a year. This points to potentially substantial positive effects on increased intake of essential nutrients through food, and on weight loss and control, as well as on various health outcomes. Based on the present results and literature, [see also e.g. (75)], we recommend 6000 transport steps per day, or equivalent, for individuals with normal weight, during five weekdays, over the year, in order to reach optimal health gains.

## Data availability statement

The raw data supporting the conclusions of this article will be made available by the authors, without undue reservation.

## Ethics statement

The studies involving human participants were reviewed and approved by the Ethics Committee North of the Karolinska Institute at the Karolinska Hospital, Stockholm, Sweden. The participants provided their written informed consent to participate in this study.

## Author contributions

PS conceived the overall aim of the study and designed it as well as drafted the main part of the manuscript. JS, HR, and PS were responsible for collecting and analyzing the data. HR and PS were responsible for the quality of the measurement devices in the laboratory and in the field, and tutored KO and JS. JS drafted the first version of the method section. KO, JS, and PS thereafter developed the manuscript in various ways. All authors read, commented, and accepted the final manuscript.

## Funding

This study was funded by the Public Health Funds of the Stockholm County Council (LS0401-0158) and the Research Funds of the Swedish Transport Administration (TRV 2017/63917-6522 and TRV 2020/119325).

## Conflict of interest

The authors declare that the research was conducted in the absence of any commercial or financial relationships that could be construed as a potential conflict of interest.

## Publisher's note

All claims expressed in this article are solely those of the authors and do not necessarily represent those of their affiliated organizations, or those of the publisher, the editors and the reviewers. Any product that may be evaluated in this article, or claim that may be made by its manufacturer, is not guaranteed or endorsed by the publisher.
