# Supplementary material for: Perspectives on exercise intensity, volume, step characteristics and health outcomes in walking for transport
Source: Front Public Health. 2022 Oct 21;10:911863. doi: 10.3389/fpubh.2022.911863 (PMC9635924; doi:10.3389/fpubh.2022.911863)
Supplement: Supplementary file 1 [file Table_1.pdf]

Supplementary File 1

This is a supplementary file to Schantz et al. (2022).

Supplementary Table 1. All variables with a significant ($p < 0.05$) difference between the sexes.

Table	**Variable** (unit)	**Absolute difference**	**Relative difference** (%)	**Effect size Hedges' correction**
1	Height (m)	0.11	6.5	1.90
	Weight (kg)	19.4	31.7	1.81
	BMI ($kg \cdot m^{-2}$)	3.5	16.1	1.22
	HR rest ($beats \cdot min^{-1}$)	13.2	21.7	1.60
	Blood pressure, diastole (mm Hg)	8.7	12.4	0.94
3	VO_2 of 1 MET ($L \cdot min^{-1}$)	0.058	31.7	1.81
	VO_2max ergometer cycling ($L \cdot min^{-1}$)	0.71	30.6	1.90
4	VO_2max treadmill running ($L \cdot min^{-1}$)	0.76	29.6	2.20
5	Walking environment*	-0.70	-77.8	-0.98
6	Step frequency in field ($steps \cdot min^{-1}$)	-7	-5.9	-1.53
7	VO_2 in field ($L \cdot min^{-1}$)	0.32	26.7	1.18
	VO_2 - 1 MET in field ($L \cdot min^{-1}$)	0.26	25.8	1.03
8	Energy expenditure in field ($kcal \cdot min^{-1}$)	1.59	27.6	1.21
	Energy expenditure in field - 1 MET ($kcal \cdot min^{-1}$)	1.30	26.7	1.06

Supplementary Table 1 (Continued). All variables with a significant ($p < 0.05$) difference between the sexes.

Table	**Variable** (unit)	Absolute difference	Relative difference (%)	Effect size Hedges' correction
9	Ventilation in field ($L \cdot min^{-1}$)	7.9	25.4	0.93
	Tidal volume in field (L)	0.41	32.6	1.52
	RPE leg in field	2.0	23.0	1.35
	RPE breathing in field	1.8	19.1	1.03
10	VO_2 in field ($L \cdot km^{-1}$)	3.8	31.6	1.23
	VO_2 in field - 1 MET ($L \cdot km^{-1}$)	3.1	30.7	1.11
11	Energy expenditure in field ($kcal \cdot km^{-1}$)	19.1	32.5	1.26
	Energy expenditure in field - 1 MET ($kcal \cdot km^{-1}$)	15.6	31.6	1.14
12	VO_2 in field ($mL \cdot step^{-1}$)	3.4	35.1	1.40
	VO_2 in field - 1 MET ($mL \cdot step^{-1}$)	2.8	34.2	1.25
	Energy expenditure in field ($cal \cdot step^{-1}$)	16.8	36.1	1.43
	Energy expenditure in field - 1 MET ($cal \cdot step^{-1}$)	13.8	35.2	1.27

Notes: *Walking environment: 0 = inner urban; 1 = inner urban – suburban; 2 = suburban.

Calculation of absolute sex difference = men's mean difference - women's mean difference.

Calculation of relative sex difference = (absolute sex difference · women's mean difference^{-1}) * 100.

According to Cohen (1988), the effect size can be interpreted as: small = 0.2, medium = 0.5, and large = 0.8.

Refrences

Cohen, J. (1988). *Statistical power analysis for the behavioral sciences.* Hillsdale, N.J.: Lawrence Erlbaum Associates.

Schantz, P., Olsson, K.S.E., Salier Eriksson, J., and Rosdahl, H. (2022). Perspectives on exercise intensity, volume, step characteristics and health outcomes in walking for transport. *Frontiers in Public Health*. 10:911863. doi: 10.3389/fpubh.2022.911863.
