# Supplementary material for: Perspectives on exercise intensity, volume, step characteristics and health outcomes in walking for transport
Source: Front Public Health. 2022 Oct 21;10:911863. doi: 10.3389/fpubh.2022.911863 (PMC9635924; doi:10.3389/fpubh.2022.911863)
Supplement: Supplementary file 2 [file Data_Sheet_1.pdf]

Supplementary File 2

This is a supplementary file to Schantz et al. (2022). The aim of it is to present data that permits comparisons between level (0.0°) treadmill walking at different speeds vs. walking in naturalistic environments and conditions for commuting purposes. The treadmill walking data consists of partially unpublished variables, which are based on individual values from two different studies; Olsson et al. (2020) and Olsson et al. (2022). They were carried out in the same laboratory using essentially the same well-controlled equipment. The naturalistic field walking refers to data from the present main study (Schantz et al. 2022).

Olsson et al. (2020) is based on the same group of participants as in the present main study (Schantz et al. 2022), i.e. habitual male and female commuting pedestrians. However, there was a reduced number of participants who completed the treadmill tests, which included three levels of walking speeds and a maximal running test. Characteristics of this group of participants (n = 16) are presented in Supplementary Table 1. Values obtained from the walking levels in terms of oxygen uptake (VO_2) and energy expenditure (EE) are given in Supplementary Table 2 and in Supplementary Figure 1 for males and females separately. All measurement procedures can be found in Olsson et al. (2020).

In Olsson et al. (2022), 24 physically active and healthy males and females participated (Supplementary Table 2), and performed six levels of walking speeds. Variables obtained from these levels in terms of step characteristics, VO_2 and EE are presented in Supplementary Table 3 and in Supplementary Figures 2 and 3 for males and females separately. The overall measurement procedures are described in Olsson et al. (2022). However, the estimations of cadence and step length were not mentioned. These were obtained by measuring the time it took to perform 20 complete steps with the right leg, which were visually observed and counted in real time by the investigator. During the measured time period, it was assumed that 20 complete steps were taken with both legs separately, and thereby the step cadence was calculated. Based on the fixed levels of speeds and the measured time periods of 20 steps with both legs, the step lengths were calculated.

Finally, the above-mentioned data on level treadmill walking from the two groups of participants based on Olsson et al. 2020 and Olsson et al. 2022 is illustrated in Supplementary Figures 4 and 5 as mean values for males and females together. In these figures, the corresponding mean values from the walking commuting in the field are displayed based on the same male and female participants (n = 16) from the present main study (Schantz et al. 2022) as in the currently used group from Olsson et al. (2020).

Supplementary Table 1. Characteristics of the participants groups based on Olsson et al. (2020) and Olsson et al. (2022) (mean ± SD).

**Study**		**Age** (years)	**Height** (m)	**Weight** (kg)	**BMI** (kg · m^{-2})
**Olsson et al. (2020)**	**Men** (n = 8)	45.3 ± 8.5	1.79 ± 0.06*	80.6 ± 12.7*	25.1 ± 3.4*
	**Women** (n = 8)	43.0 ± 4.7	1.68 ± 0.05*	60.6 ± 9.3*	21.3 ± 2.7*
**Olsson et al. (2022)**	**Men** (n = 12)	28.8 ± 7.9	1.83 ± 0.09*	81.4 ± 9.1*	24.1 ± 1.0*
	**Women** (n = 12)	29.4 ± 8.1	1.66 ± 0.07*	60.2 ± 5.1*	21.9 ± 1.5*

Notes: BMI = body mass index.

* denotes a significant sex difference ($p < 0.05$) within each study, which has been calculated with the independent T-test.

Supplementary Table 2. VO_2 and EE per body weight and unit of time or distance in level treadmill walking based on Olsson et al. (2020) (mean ± SD and 95% CI).

**Participants**	**Speed** (km · h^{-1}):	**4.0**	**4.9**	**5.9**
**Men**	**VO$_2$** (mL · kg^{-1} · min^{-1})	10.2 ± 1.0	12.1 ± 1.2	15.0 ± 1.8
(n = 8)		(9.4 – 11.0)	(11.1 – 13.1)	(13.5 – 16.5)
	***Relative change** (%)*	*100*	*119 ± 13 (108 – 130)*	*148 ± 19 (132 – 164)*
	**VO$_2$** (L · km^{-1})	12.2 ± 1.2*	12.0 ± 2.3*	12.4 ± 2.6*
		(11.2 – 13.2)	(10.0 – 13.9)	(10.2 – 14.5)
	***Relative change** (%)*	*100*	*97 ± 11 (88 – 106)*	*100 ± 13 (89 – 111)*
	**VO$_2$** (mL · kg^{-1} · km^{-1})	153 ± 15	148 ± 15	153 ± 18
		(141 – 165)	(136 – 160)	(137 – 168)
	***Relative change** (%)*	*100*	*97 ± 11 (88 – 106)*	*100 ± 13 (89 – 111)*
	**EE** (kcal · kg^{-1} · min^{-1})	0.0499 ± 0.0050	0.0590 ± 0.0058	0.0733 ± 0.0089
		(0.0457 – 0.0541)	(0.0542 – 0.0638)	(0.0658 – 0.0807)
	***Relative change** (%)*	*100*	*119 ± 13 (108 – 130)*	*148 ± 20 (131 – 165)*
	**EE** (kcal · km^{-1})	59.7 ± 5.6*	58.4 ± 11.0*	60.3 ± 12.5*
		(55.0 – 64.4)	(49.2 – 67.5)	(49.8 – 70.7)
	***Relative change** (%)*	*100*	*97 ± 11 (88 – 106)*	*100 ± 14 (89 – 112)*
	**EE** (kcal · kg^{-1} · km^{-1})	0.749 ± 0.075	0.723 ± 0.071	0.745 ± 0.090
		(0.686 – 0.811)	(0.664 – 0.782)	(0.670 – 0.821)
	***Relative change** (%)*	*100*	*97 ± 11 (88 – 106)*	*100 ± 14 (89 – 112)*
**Participants**	**Speed** (km · h^{-1}):	**4.0**	**4.9**	**5.9**
**Women**	**VO$_2$** (mL · kg^{-1} · min^{-1})	10.1 ± 0.7	12.3 ± 0.8	14.9 ± 1.2
(n = 8)		(9.5 – 10.7)	(11.7 – 12.9)	(13.9 – 15.9)
	***Relative change** (%)*	*100*	*121 ± 5 (117 – 125)*	*148 ± 13 (137 – 159)*
	**VO$_2$** (L · km^{-1})	9.2 ± 1.5*	9.1 ± 1.4*	9.2 ± 1.5*
		(7.9 – 10.5)	(7.9 – 10.3)	(8.0 – 10.4)
	***Relative change** (%)*	*100*	*99 ± 4 (96 – 102)*	*100 ± 9 (93 – 108)*
	**VO$_2$** (mL · kg^{-1} · km^{-1})	152 ± 11	151 ± 9	152 ± 12
		(143 – 161)	(143 – 158)	(142 – 162)
	***Relative change** (%)*	*100*	*99 ± 4 (96 – 102)*	*100 ± 9 (93 – 108)*
	**EE** (kcal · kg^{-1} · min^{-1})	0.0493 ± 0.0035	0.0598 ± 0.0036	0.0730 ± 0.0061
		(0.0464 – 0.0522)	(0.0568 – 0.0628)	(0.0679 – 0.0781)
	***Relative change** (%)*	*100*	*122 ± 5 (117 – 126)*	*149 ± 13 (138 – 160)*
	**EE** (kcal · km^{-1})	44.8 ± 7.8*	44.4 ± 7.3*	44.9 ± 7.5*
		(38.3 – 51.3)	(38.3 – 50.5)	(38.6 – 51.2)
	***Relative change** (%)*	*100*	*99 ± 4 (96 – 103)*	*101 ± 9 (93 – 108)*
	**EE** (kcal · kg^{-1} · km^{-1})	0.739 ± 0.052	0.733 ± 0.044	0.743 ± 0.062
		(0.696 – 0.783)	(0.696 – 0.769)	(0.691 – 0.795)
	***Relative change** (%)*	*100*	*99 ± 4 (96 – 103)*	*101 ± 9 (93 – 108)*

Notes: The relative changes are based on the first speed level (4 km · h^{-1}) for each variable and sex.

* denotes a significant sex difference ($p < 0.05$), which has been calculated with the independent T-test.

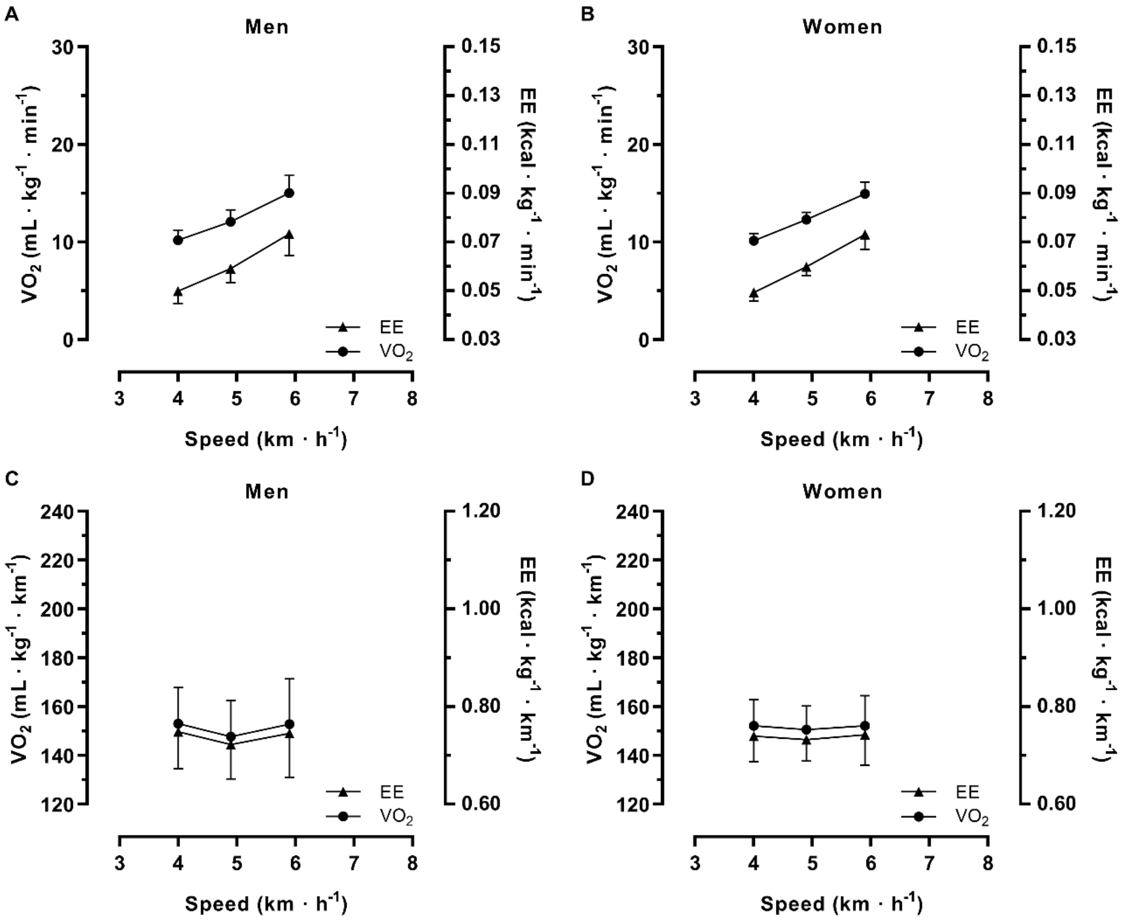

Supplementary Figure 1. VO_2 and EE per body weight and unit of time (**A**, **B**) or distance (**C**, **D**) for males (n = 8) and females (n = 8) at fixed speeds of level treadmill walking based on Olsson et al. (2020). In mean values ± SD.

Supplementary Table 3. Step characteristics, VO_2 and EE per body weight and unit of time or distance in level treadmill walking based on Olsson et al. (2022) (mean ± SD and 95% CI).

Participants	Speed (km · h^{-1}):	3.0	4.0	5.0	6.0	7.0	8.0
**Men** (n = 12)	**Step length** (m)	0.615 ± 0.039* (0.591 – 0.640)	0.698 ± 0.032* (0.678 – 0.718)	0.795 ± 0.029* (0.776 – 0.813)	0.872 ± 0.029* (0.853 – 0.890)	0.946 ± 0.033* (0.925 – 0.968)	0.997 ± 0.031* (0.976 – 1.018)
	***Relative change** (%)*	*100*	*114 ± 4 (111 – 116)*	*129 ± 5 (126 – 133)*	*142 ± 7 (138 – 147)*	*154 ± 8 (149 – 159)*	*160 ± 12 (152 – 169)*
	**Cadence** (step · min^{-1})	82 ± 6* (78 – 85)	96 ± 4* (93 – 98)	105 ± 4* (102 – 108)	115 ± 4* (112 – 117)	123 ± 5* (120 – 126)	134 ± 4* (131 – 137)
	***Relative change** (%)*	*100*	*117 ± 4 (115 – 120)*	*129 ± 5 (126 – 132)*	*141 ± 7 (137 – 146)*	*152 ± 8 (147 – 157)*	*167 ± 12 (159 – 175)*
	**VO_2** (mL · kg^{-1} · min^{-1})	9.2 ± 0.6* (8.8 – 9.6)	10.5 ± 0.8 (10.0 – 11.0)	12.3 ± 0.7 (11.9 – 12.8)	15.4 ± 1.3 (14.5 – 16.2)	19.9 ± 1.6 (18.8 – 20.9)	25.7 ± 1.8 (24.5 – 26.9)
	***Relative change** (%)*	*100*	*114 ± 4 (111 – 116)*	*134 ± 6 (130 – 138)*	*167 ± 9 (161 – 173)*	*216 ± 10 (209 – 222)*	*282 ± 19 (270 – 295)*
	**VO_2** (mL · kg^{-1} · step^{-1})	0.113 ± 0.007* (0.109 – 0.117)	0.109 ± 0.008* (0.104 – 0.114)	0.118 ± 0.007* (0.113 – 0.122)	0.134 ± 0.010* (0.127 – 0.140)	0.161 ± 0.013* (0.153 – 0.169)	0.192 ± 0.013 (0.184 – 0.200)
	***Relative change** (%)*	*100*	*97 ± 5 (94 – 100)*	*104 ± 5 (101 – 107)*	*118 ± 9 (113 – 124)*	*142 ± 8 (137 – 148)*	*169 ± 10 (163 – 176)*
	**VO_2** (L · km^{-1})	14.9 ± 1.4* (14.0 – 15.8)	12.7 ± 1.2* (11.9 – 13.5)	12.0 ± 1.0* (11.3 – 12.7)	12.4 ± 0.8* (11.9 – 12.9)	13.8 ± 1.1* (13.1 – 14.5)	16.0 ± 1.0* (15.3 – 16.7)
	***Relative change** (%)*	*100*	*85 ± 3 (83 – 87)*	*80 ± 4 (78 – 83)*	*83 ± 5 (80 – 86)*	*92 ± 4 (90 – 95)*	*106 ± 7 (101 – 110)*
	**VO_2** (mL · kg^{-1} · km^{-1})	184 ± 12* (176 – 192)	157 ± 12 (149 – 164)	148 ± 8 (143 – 153)	154 ± 13 (145 – 162)	170 ± 14 (161 – 179)	193 ± 13 (184 – 202)
	***Relative change** (%)*	*100*	*85 ± 3 (83 – 87)*	*80 ± 4 (78 – 83)*	*83 ± 5 (80 – 86)*	*92 ± 4 (90 – 95)*	*106 ± 7 (101 – 110)*
	**EE** (kcal · kg^{-1} · min^{-1})	0.0441 ± 0.0029* (0.0422 – 0.0459)	0.0506 ± 0.0035 (0.0483 – 0.0528)	0.0600 ± 0.0037 (0.0576 – 0.0624)	0.0749 ± 0.0067 (0.0706 – 0.0791)	0.0970 ± 0.0083 (0.0918 – 0.1023)	0.1264 ± 0.0086 (0.1206 – 0.1323)
	***Relative change** (%)*	*100*	*115 ± 4 (112 – 117)*	*136 ± 6 (132 – 140)*	*170 ± 10 (163 – 176)*	*220 ± 10 (214 – 226)*	*290 ± 18 (278 – 302)*
	**EE** (cal · kg^{-1} · step^{-1})	0.541 ± 0.036* (0.518 – 0.564)	0.529 ± 0.036* (0.506 – 0.552)	0.572 ± 0.038* (0.548 – 0.596)	0.652 ± 0.050* (0.620 – 0.683)	0.786 ± 0.063* (0.747 – 0.826)	0.945 ± 0.061 (0.904 – 0.986)
	***Relative change** (%)*	*100*	*98 ± 5 (95 – 101)*	*106 ± 5 (103 – 109)*	*121 ± 9 (115 – 126)*	*145 ± 9 (140 – 151)*	*174 ± 10 (167 – 181)*
	**EE** (kcal · km^{-1})	71.5 ± 6.4* (67.4 – 75.6)	61.5 ± 5.6* (57.9 – 65.0)	58.4 ± 5.0* (55.2 – 61.6)	60.5 ± 3.6* (58.2 – 62.7)	67.3 ± 5.1* (64.0 – 70.5)	78.7 ± 4.8* (75.5 – 81.9)
	***Relative change** (%)*	*100*	*86 ± 3 (84 – 88)*	*82 ± 4 (79 – 84)*	*85 ± 5 (82 – 88)*	*94 ± 4 (92 – 97)*	*109 ± 7 (104 – 113)*
	**EE** (kcal · kg^{-1} · km^{-1})	0.882 ± 0.058* (0.845 – 0.919)	0.758 ± 0.052 (0.725 – 0.791)	0.720 ± 0.045 (0.692 – 0.749)	0.749 ± 0.067 (0.706 – 0.791)	0.832 ± 0.071 (0.787 – 0.877)	0.948 ± 0.065 (0.905 – 0.992)
	***Relative change** (%)*	*100*	*86 ± 3 (84 – 88)*	*82 ± 4 (79 – 84)*	*85 ± 5 (82 – 88)*	*94 ± 4 (92 – 97)*	*109 ± 7 (104 – 113)*

The table continues on the next page.

Supplementary Table 3 (Continued). Step characteristics, VO_2 and EE per body weight and unit of time or distance in level treadmill walking based on Olsson et al. (2022) (mean ± SD and 95% CI).

Participants	Speed (km · h^{-1}):	3.0	4.0	5.0	6.0	7.0	8.0
**Women** (n = 12)	**Step length** (m)	0.568 ± 0.047* (0.539 – 0.598)	0.649 ± 0.043* (0.622 – 0.676)	0.734 ± 0.042* (0.708 – 0.761)	0.810 ± 0.045* (0.782 – 0.838)	0.869 ± 0.044* (0.841 – 0.896)	0.894 ± 0.050* (0.863 – 0.926)
	***Relative change** (%)*	*100*	*114 ± 4 (112 – 117)*	*130 ± 6 (126 – 133)*	*143 ± 6 (139 – 147)*	*153 ± 9 (148 – 159)*	*158 ± 9 (152 – 163)*
	**Cadence** (step · min^{-1})	89 ± 7* (84 – 93)	103 ± 7* (99 – 107)	114 ± 6* (110 – 118)	124 ± 7* (119 – 128)	135 ± 7* (130 – 139)	149 ± 8* (144 – 155)
	***Relative change** (%)*	*100*	*117 ± 4 (114 – 119)*	*129 ± 5 (125 – 132)*	*140 ± 6 (136 – 144)*	*153 ± 9 (147 – 158)*	*169 ± 9 (163 – 175)*
	**VO_2** (mL · kg^{-1} · min^{-1})	8.3 ± 1.2* (7.5 – 9.1)	9.9 ± 1.2 (9.1 – 10.7)	12.0 ± 1.3 (11.2 – 12.9)	15.1 ± 1.4 (14.2 – 16.0)	20.1 ± 1.3 (19.3 – 20.9)	27.3 ± 2.1 (26.0 – 28.7)
	***Relative change** (%)*	*100*	*120 ± 6 (116 – 124)*	*147 ± 13 (138 – 155)*	*185 ± 19 (173 – 197)*	*246 ± 30 (227 – 265)*	*336 ± 51 (304 – 369)*
	**VO_2** (mL · kg^{-1} · step^{-1})	0.094 ± 0.016* (0.084 – 0.105)	0.096 ± 0.013* (0.088 – 0.105)	0.106 ± 0.012* (0.098 – 0.114)	0.123 ± 0.012* (0.115 – 0.130)	0.149 ± 0.009* (0.144 – 0.155)	0.183 ± 0.012 (0.175 – 0.191)
	***Relative change** (%)*	*100*	*103 ± 7 (99 – 107)*	*114 ± 11 (107 – 121)*	*132 ± 14 (123 – 141)*	*162 ± 22 (148 – 176)*	*199 ± 30 (180 – 218)*
	**VO_2** (L · km^{-1})	10.0 ± 1.7* (8.9 – 11.0)	8.9 ± 1.3* (8.1 – 9.7)	8.7 ± 1.0* (8.0 – 9.3)	9.1 ± 1.1* (8.4 – 9.8)	10.4 ± 1.0* (9.7 – 11.0)	12.3 ± 1.3* (11.5 – 13.1)
	***Relative change** (%)*	*100*	*90 ± 5 (87 – 93)*	*88 ± 8 (83 – 93)*	*92 ± 10 (86 – 98)*	*106 ± 13 (97 – 114)*	*126 ± 19 (114 – 138)*
	**VO_2** (mL · kg^{-1} · km^{-1})	166 ± 25* (150 – 181)	148 ± 19 (136 – 160)	144 ± 15 (135 – 154)	151 ± 14 (142 – 160)	172 ± 11 (165 – 179)	205 ± 16 (195 – 215)
	***Relative change** (%)*	*100*	*90 ± 5 (87 – 93)*	*88 ± 8 (83 – 93)*	*92 ± 10 (86 – 98)*	*106 ± 13 (97 – 114)*	*126 ± 19 (114 – 138)*
	**EE** (kcal · kg^{-1} · min^{-1})	0.0396 ± 0.0059* (0.0359 – 0.0434)	0.0478 ± 0.0060 (0.0440 – 0.0516)	0.0584 ± 0.0061 (0.0546 – 0.0623)	0.0736 ± 0.0067 (0.0693 – 0.0779)	0.0984 ± 0.0061 (0.0945 – 0.1023)	0.1346 ± 0.0107 (0.1278 – 0.1414)
	***Relative change** (%)*	*100*	*121 ± 6 (117 – 125)*	*149 ± 13 (140 – 158)*	*188 ± 19 (176 – 200)*	*252 ± 31 (233 – 272)*	*346 ± 55 (311 – 382)*
	**EE** (cal · kg^{-1} · step^{-1})	0.450 ± 0.079* (0.400 – 0.501)	0.465 ± 0.065* (0.424 – 0.506)	0.515 ± 0.059* (0.477 – 0.552)	0.596 ± 0.059* (0.558 – 0.633)	0.731 ± 0.039* (0.706 – 0.757)	0.901 ± 0.064 (0.860 – 0.942)
	***Relative change** (%)*	*100*	*104 ± 7 (100 – 108)*	*116 ± 11 (109 – 123)*	*134 ± 15 (125 – 144)*	*166 ± 23 (152 – 181)*	*205 ± 33 (184 – 226)*
	**EE** (kcal · km^{-1})	47.6 ± 8.0* (42.5 – 52.7)	43.1 ± 6.1* (39.3 – 47.0)	42.1 ± 4.9* (39.0 – 45.2)	44.2 ± 5.0* (41.1 – 47.4)	50.7 ± 4.5* (47.8 – 53.6)	60.7 ± 6.4* (56.6 – 64.7)
	***Relative change** (%)*	*100*	*91 ± 5 (88 – 94)*	*89 ± 8 (84 – 95)*	*94 ± 10 (88 – 100)*	*108 ± 13 (100 – 117)*	*130 ± 21 (117 – 143)*
	**EE** (kcal · kg^{-1} · km^{-1})	0.792 ± 0.118* (0.717 – 0.867)	0.718 ± 0.090 (0.661 – 0.775)	0.701 ± 0.073 (0.655 – 0.748)	0.736 ± 0.067 (0.693 – 0.779)	0.844 ± 0.053 (0.810 – 0.877)	1.009 ± 0.081 (0.958 – 1.061)
	***Relative change** (%)*	*100*	*91 ± 5 (88 – 94)*	*89 ± 8 (84 – 95)*	*94 ± 10 (88 – 100)*	*108 ± 13 (100 – 117)*	*130 ± 21 (117 – 143)*

Notes: The relative changes are based on the first speed level (3 km · h^{-1}) for each variable and sex.

* denotes a significant sex difference ($p < 0.05$), which has been calculated with the independent T-test.

Only 11 male participants performed the last speed level (8 km · h^{-1}).

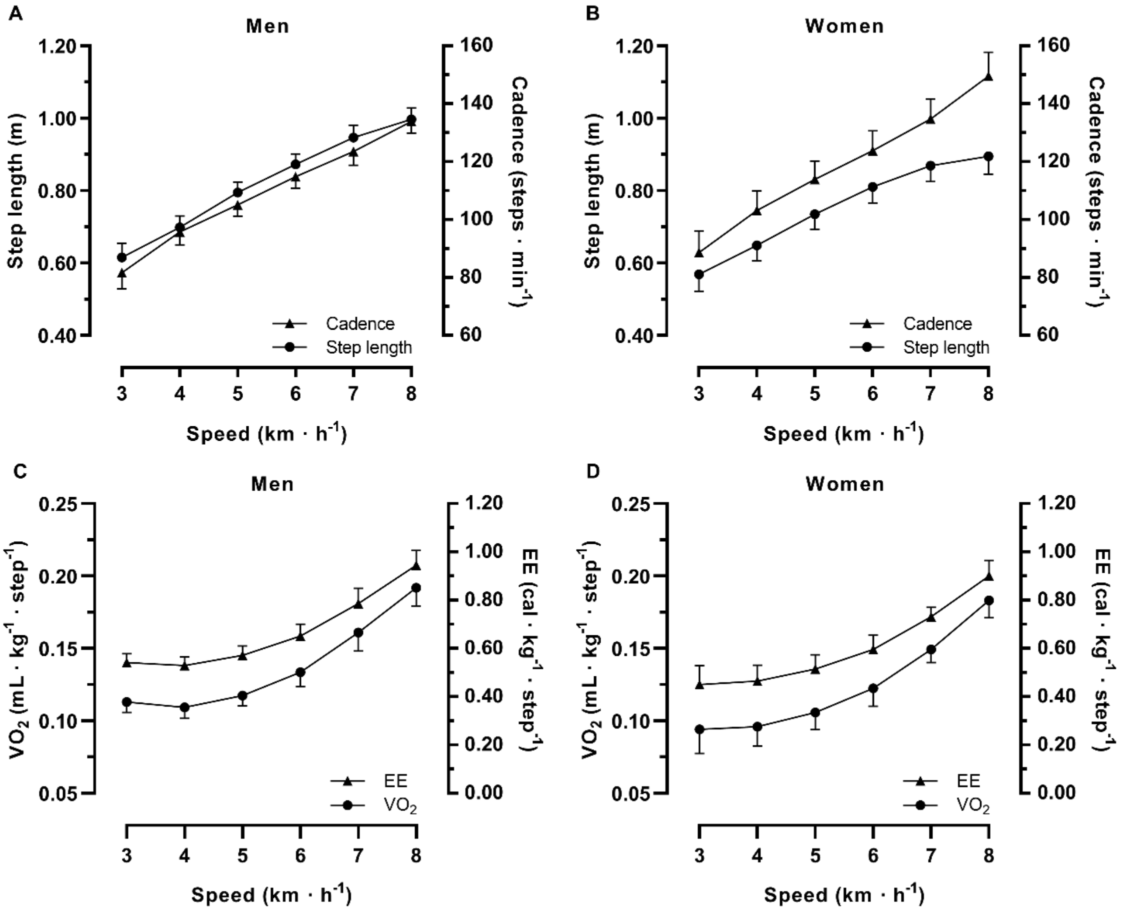

Supplementary Figure 2. Step length and cadence (**A**, **B**), and VO_2 and EE per body weight and step (**C**, **D**) for males (n = 12, only 11 males performed 8 km · h^{-1}) and females (n =12) at fixed speeds of level treadmill walking based on Olsson et al. (2022). In mean values ± SD.

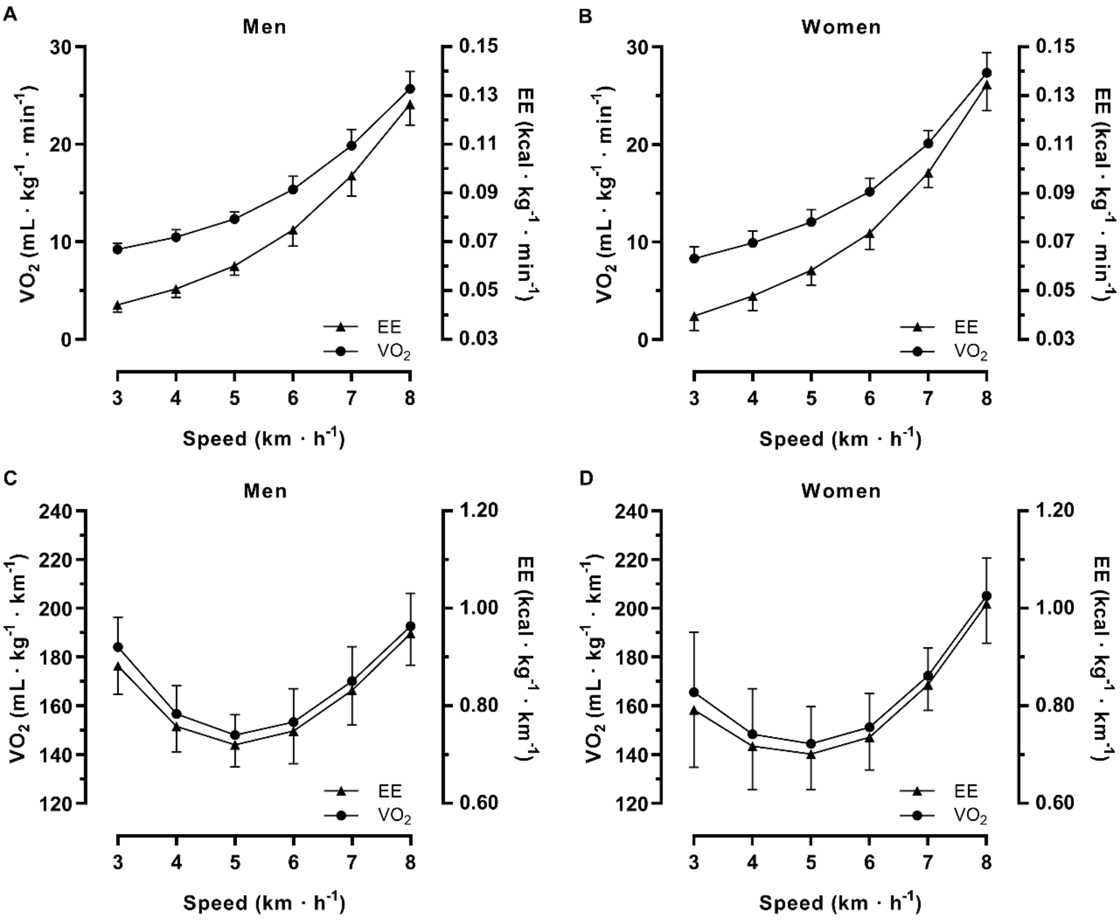

Supplementary Figure 3. VO_2 and EE per body weight and unit of time (**A**, **B**) or distance (**C**, **D**) for males (n = 12, only 11 males performed 8 km · h^{-1}) and females (n =12) at fixed speeds of level treadmill walking based on Olsson et al. (2022). In mean values ± SD.

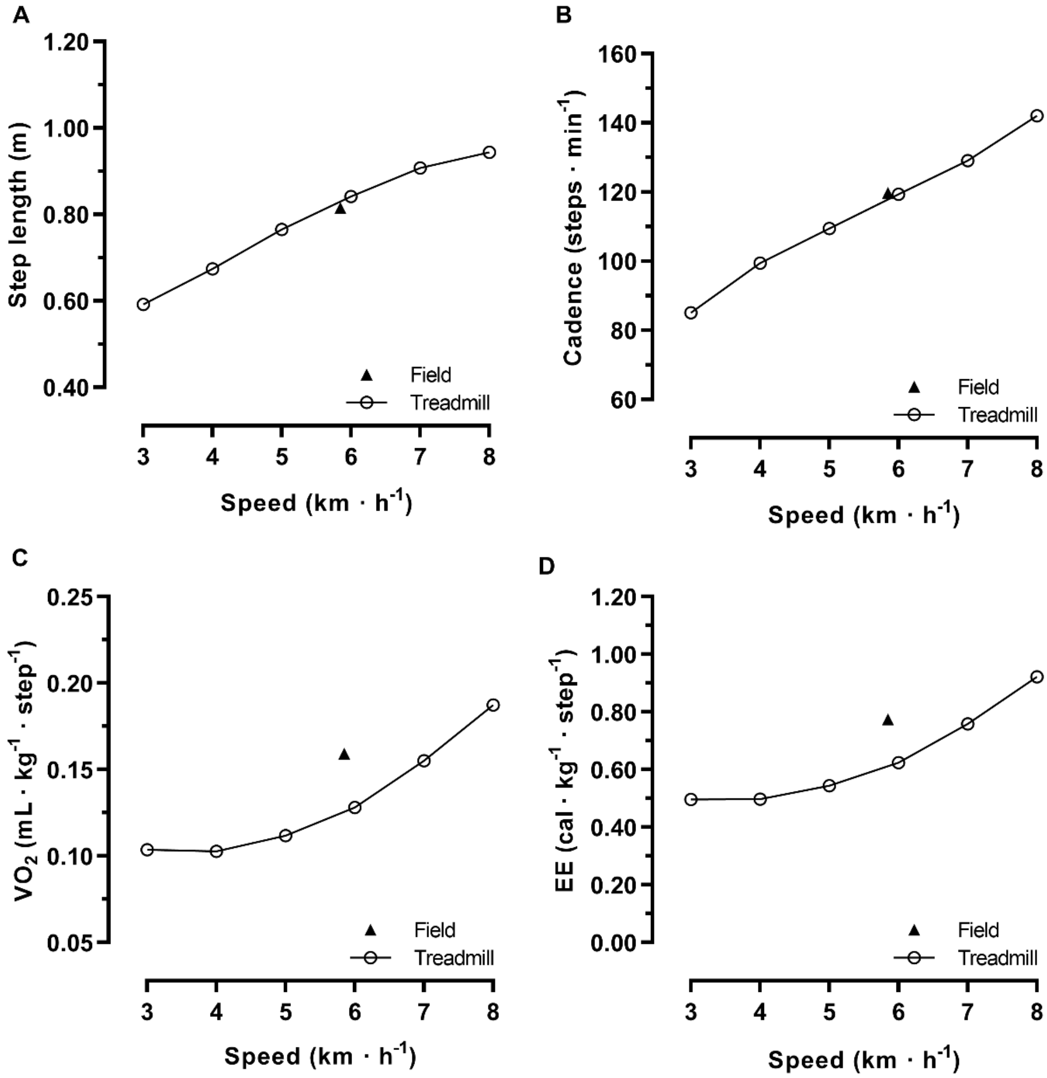

Supplementary Figure 4. Step length (**A**), cadence (**B**), and VO_2 and EE per body weight and step (**C**, **D**) during different walking conditions. Filled triangles represent mean values of walking commuting in the field of the male and female pedestrians (n = 16) in the present main study (Schantz et al. 2022). Open circles represent mean values of level treadmill walking based on the males and females (n = 24; only 23 participants performed 8 km · h^{-1}) in Olsson et al. (2022).

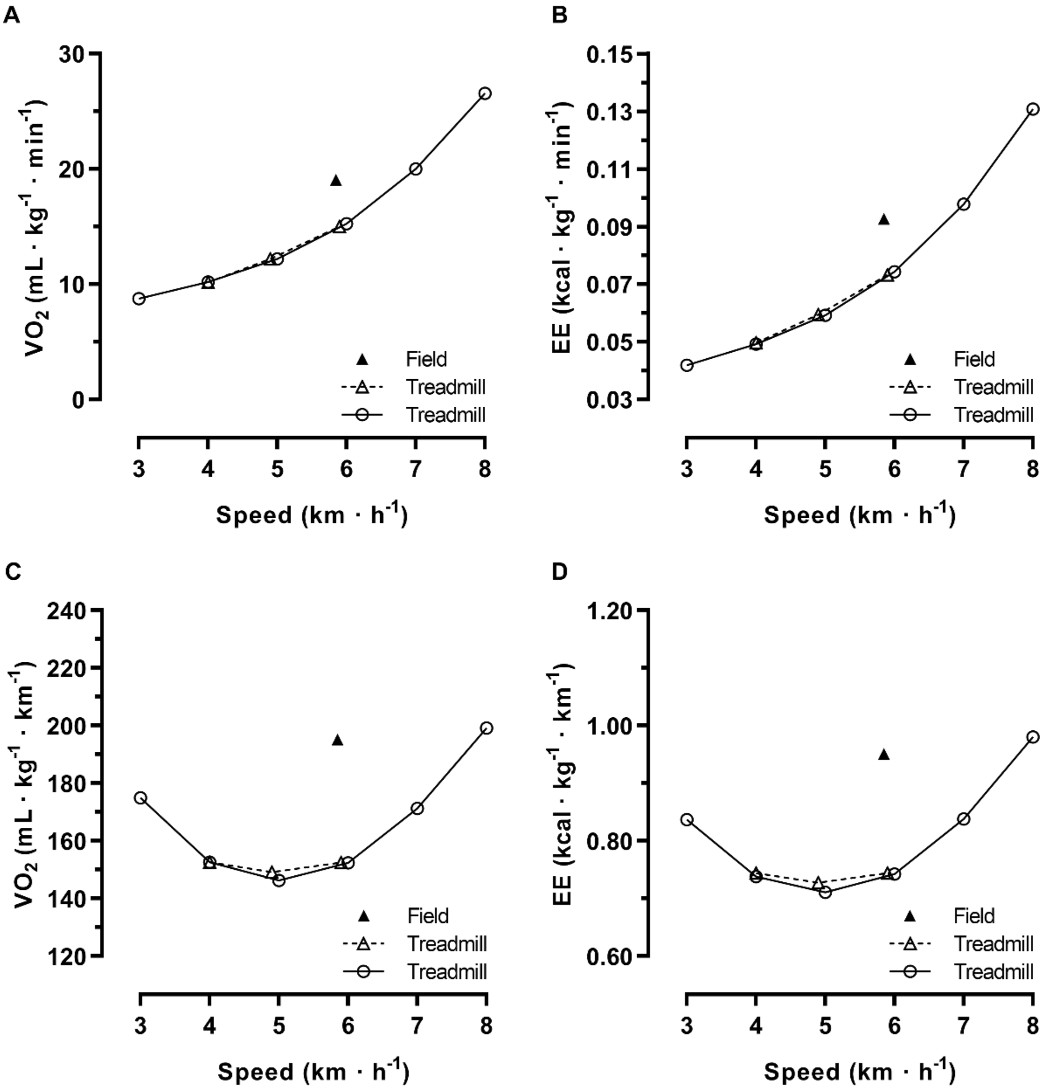

Supplementary Figure 5. VO_2 and EE per body weight and unit of time (**A**, **B**) or distance (**C**, **D**) during different walking conditions. Filled triangles represent mean values of walking commuting in the field of the male and female pedestrians (n = 16) in the present main study (Schantz et al. 2022). Open triangles represent mean values of level treadmill walking based on the male and female pedestrians (n = 16) in Olsson et al. (2020), i.e. the same group of participants as included in the present main study (Schantz et al. 2022). Open circles represent mean values of level treadmill walking based on the males and females (n = 24; only 23 participants performed 8 km · h^{-1}) in Olsson et al. (2022).

References

Olsson, K., Salier Eriksson, J., Rosdahl, H., and Schantz, P. (2020). Are heart rate methods based on ergometer cycling and level treadmill walking interchangeable? *PLOS ONE* 15**,** e0237388.

Olsson, K.S.E., Rosdahl, H., and Schantz, P. (2022). Interchangeability and optimization of heart rate methods for estimating oxygen uptake in ergometer cycling, level treadmill walking and running. *BMC Medical Research Methodology* 22**,** 55.

Schantz, P., Olsson, K.S.E., Salier Eriksson, J., and Rosdahl, H. (2022). Perspectives on exercise intensity, volume, step characteristics and health outcomes in walking for transport. *Frontiers in Public Health*. 10:911863. doi: 10.3389/fpubh.2022.911863.
